# A mobile app for Glaucoma diagnosis and its possible clinical applications

**DOI:** 10.1186/s12911-020-1123-2

**Published:** 2020-07-09

**Authors:** Fan Guo, Weiqing Li, Xin Zhao, Junfeng Qiu, Yuxiang Mai

**Affiliations:** School of Automation, Central South University, Changsha, China

**Keywords:** Mobile, E-health, Glaucoma, App development, Clinical application

## Abstract

**Background:**

Nowadays, the latent power of technology, which can offer innovative resolutions to disease diagnosis, has awakened high-level anticipation in the community of patients as well as professionals. An easy-to-use mobile app is developed by us, which is purposefully intended for those patients with glaucoma.

**Methods:**

A mobile App has been invented for smartphones for the convenient use wherever and whenever. The corresponding experiments carried out by public retinal image database and real captured clinical data reveal the ideal classification accuracy of the App. Also, user feedback evaluation is also carried out in terms of performance test as well as and users’ experience.

**Results:**

For clinical test using Yanbao App, we found 274 patients for the identification with 648 retinal images to be evaluated by glaucoma classification. Of the 243 glaucoma patients, 191 were screened out with an accuracy of 0.7860 (sensitivity); the number of non-glaucoma patients was 310 of 405, and the accuracy reached 0.7654 (specificity).` The total Accuracy amounted to 0.7731, and the result is close to the test performance obtained on public dataset ORIGA and DRISHTI-GS1.

**Conclusions:**

Yanbao App can be applied as an innovative approach exploiting mobile technology to enhance the clinicians’ efficiency and a balanced medical resources as well as a provided better tiered medical service system.

## Background

E-health technologies are ever considered as high potential tools to enhance healthcare quality, availability as well as the delivery. Recent years have seen that the smart technology is featured by the raising high-level potential to offer high expectation innovative resolutions for disease treatment for the community of patients and healthcare professionals [1]. Therefore, many Apps have appeared. They are of great help to manage chronic conditions [2]. Some Apps are equipped with a broad spectrum on the generalized medical knowledge; others are made for special purposes.

A mobile App called Yanbao, is presented in this thesis, which is designed for those who are diagnosed with glaucoma. Glaucoma, which is billed as the second major blindness cause is meanwhile the foremost pathogeny of the irreversible blindness. Despite that no cure for the disease, early detection and treatment are able to lessen the blindness rate. Thereby, this easy-to-use App can bring practical advantage to most of the users. The job dealt with the difficulty in virtue of bringing forward a new App which offers a good opportunity to users to for convenient glaucoma diagnosis. The mobile App is billed as a technological method which helps the glaucoma patients defeat illness-correlated issues and reduce their thought burden as they can allow the patients to share the service of high-quality screen whenever and wherever. Accordingly, the mobile App can enhance the clinic efficiency, strike optimize medical resources by providing better services.

The existing works related to our work can be classified into two categories: solutions for glaucoma screening discussed, and applications which help users tackle their health and fitness. In terms of large scale glaucoma screening, color fundus images are of suitability as a result of the cost effectiveness. Accordingly, a number of works been conducted to automated glaucoma detection system in virtue of analyzing the input retinal images. For example, Soltani et al. [3] carried out an updated diagnostic system, aiming to draw out the parameters vital to the glaucoma screening. Using novel blood vessel tracking as well as bending point detection, Soorya et al. [4] put forward an automated algorithm for glaucoma diagnosis based on retinal images. Sousa et al. [5] came up with a method analyzing the texture of optical disk (OD) region for glaucoma diagnosis. Fu et al. [6] set up a Disc-aware Ensemble Network (DENet) for glaucoma diagnosis. That combined four deep streams at different levels. The multiple levels and modules offer big benefit to comprise the hierarchical representations; in the meanwhile, the disc-aware constraint ensures the contextual information based on the OD region for the screening of glaucoma. Chai et al. [7] raised and incorporated the domain knowledge to structure a two-branch Convolutional Neural Networks (CNN), in order to gain the learning on a classifier on the basis of the retinal image [8] provided that different classification methods have been used up to now for glaucoma detection. Besides, the paper also reaches the conclusion that employing SVM classifier to detect normal and abnormal cases can give rise to satisfying performance. In conclusion, glaucoma diagnosis is inclined to call for some major clinical parameters as well as a good classifier. An App can be beneficial to those who intend to obtain optimal clinical parameter values as well as the probability of coming down with glaucoma.

On the other hand, the mobile Apps is popularized as a commonly used device in medical area. For example, Ricci et al. [1] carried out a mobile App, AIGkit, which is purposefully schemed for adult patients with Pompe illness. Other Apps, like the MOST-96120 program, were employed to decide the early cognitive impairments from dementia. The iPad App program offers the sickness scores in virtue of making the elderly patients press or draw on the surface of the Apps [9]. OphthalDSS, a mobile App tool, helps the corresponding medical students to conduct clinical decisions on pink eye disease [10]. Patterson et al. [11], who schemed an App to diagnose and predict epilepsy, which allows community health workers to conduct diseases screen as doctors do. According to [12], a mobile App tool to diagnose the illnesses of tongue images is offered. Another iOS program is schemed for the prevention of cardiovascular illness. Further, the program is able to forecast disease status from sensors installed in the human body [13]. Also, an iOS program has been implemented to enhance the patients’ reading with macular degeneration by way of dynamic text display [14]. Nonetheless these works can never clearly provide an App to let users for glaucoma diagnosis. A new App, which can fill in this literature gap, will be introduced in the nest part.

## Methods

### Hardware detail

Our Yanbao App can be used with a smartphone fundus camera, which called oDocs Fundus [15]. The camera is designed by oDocs Eye Care and it becomes the most widely download smartphone based retinal imaging adapter. It is now available as a DIY Kit or a fully assembled kit.

**Fig. 1:**
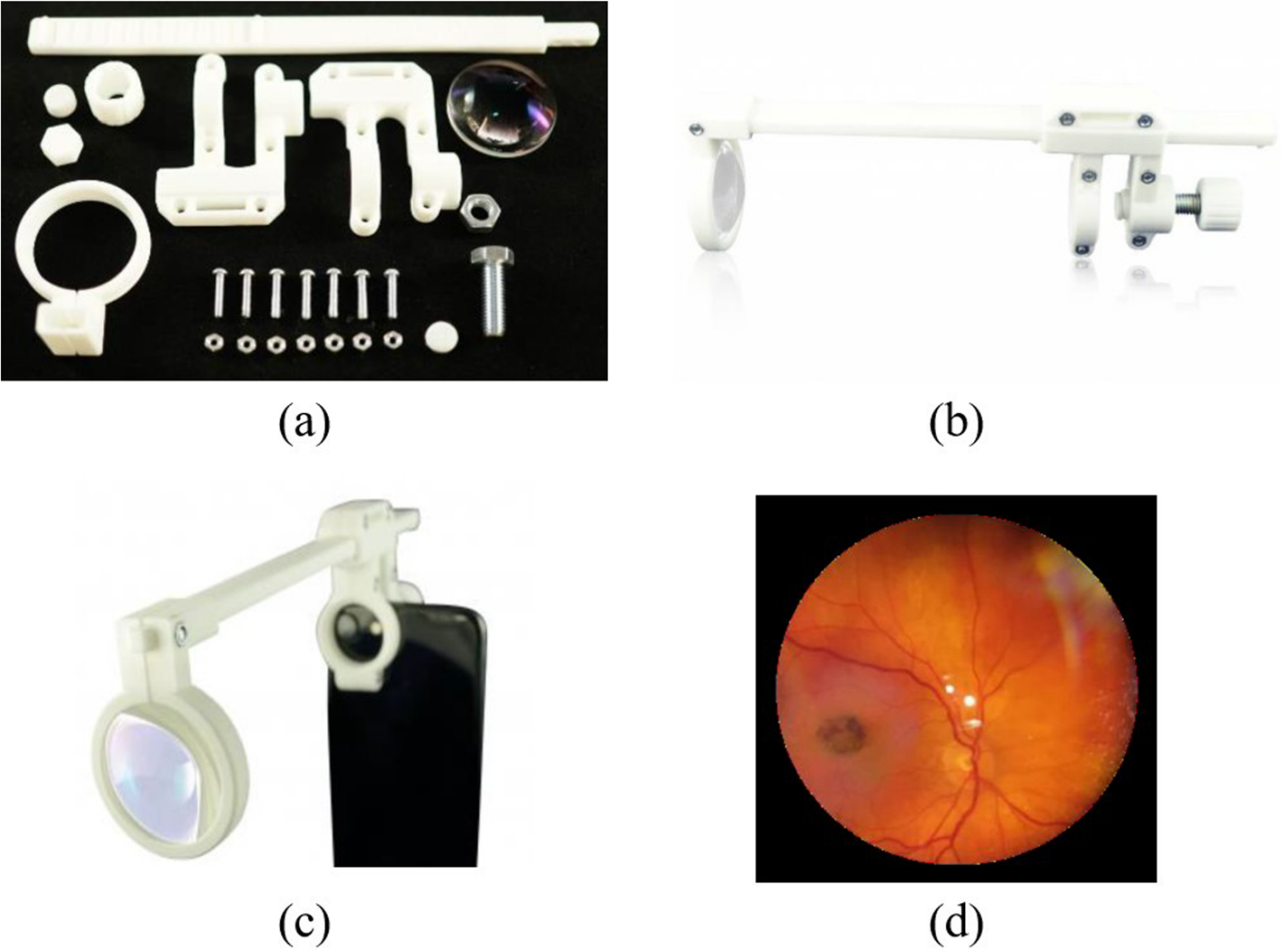
Smartphone fundus camera

Since oDocs Fundus is designed to fit a large range of iPhone and Android smartphones, users can thus conveniently capture retinal images at any time and any place. The computer aided design (CAD) files are available for 3D printing, Fig. 1(a) displays the camera parts, and the assembled smartphone ophthalmoscope / retinal imaging adapter is shown in Fig. 1(b). Combined with the smartphone, the smartphone ophthalmoscope can capture a good retinal image, as shown in Fig. 1(c) and Fig. 1(d).

### Algorithm detail

**Fig. 2:**
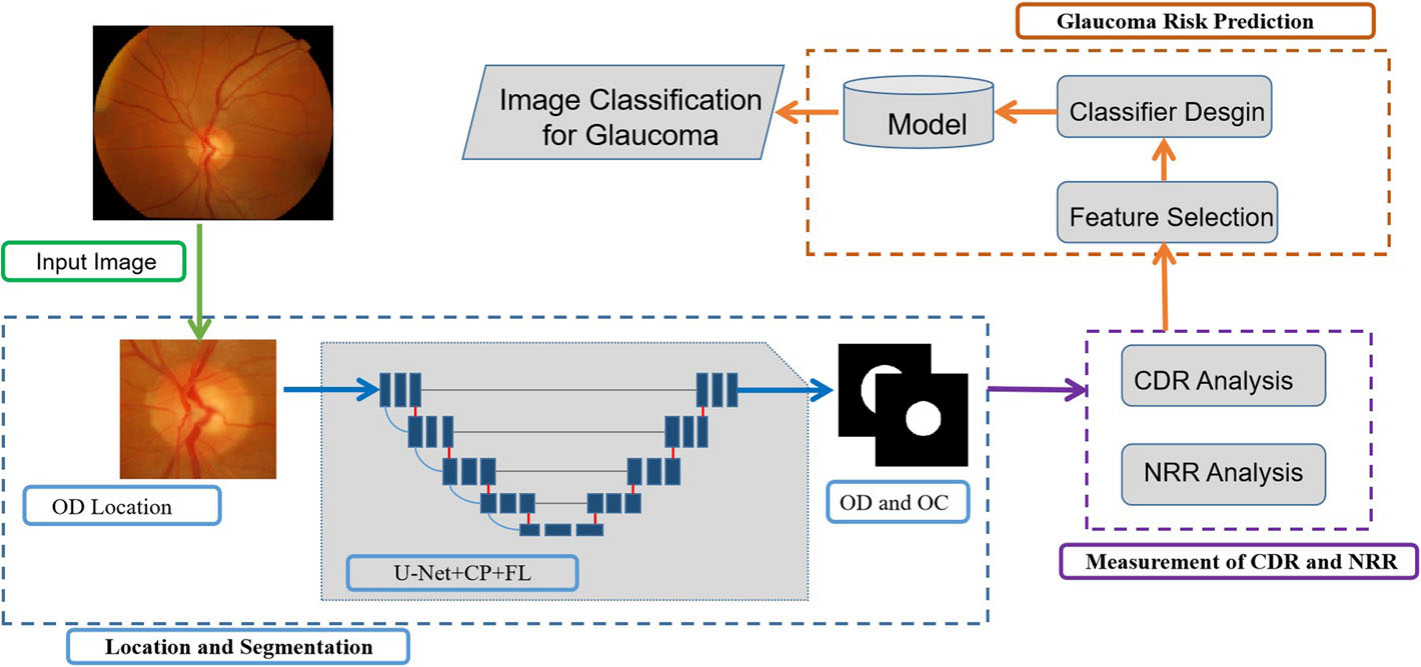
Flowchart of the internal algorithm of Yanbao

Figure 2 illustrates the internal algorithm of Yanbao’s framework, which is made up of two major phases: (a) ROI Localization and optical disk (OD) and cup (OC) segmentation; (b) CDR and NRR measurement as well as glaucoma classification.

As Fig. 2 indicates, a new optic disc positioning algorithm is first proposed and the OD is localized from an image. Next, segment OD and OC based on the background are put forward by the suggested novel network U-Net + CP + FL, performing performs better than U-Net. Next, the measurement to CDR and NRR is conducted to conclude the likeliness of glaucoma. For instance, the higher CDR conforms to a higher glaucoma risk. Eventually, the glaucoma classification stage categories the test image of the normal from glaucoma. The next subsections will discuss the subtasks which the block diagram has to shoulder.

#### Localization and segmentation

**Fig. 3:**
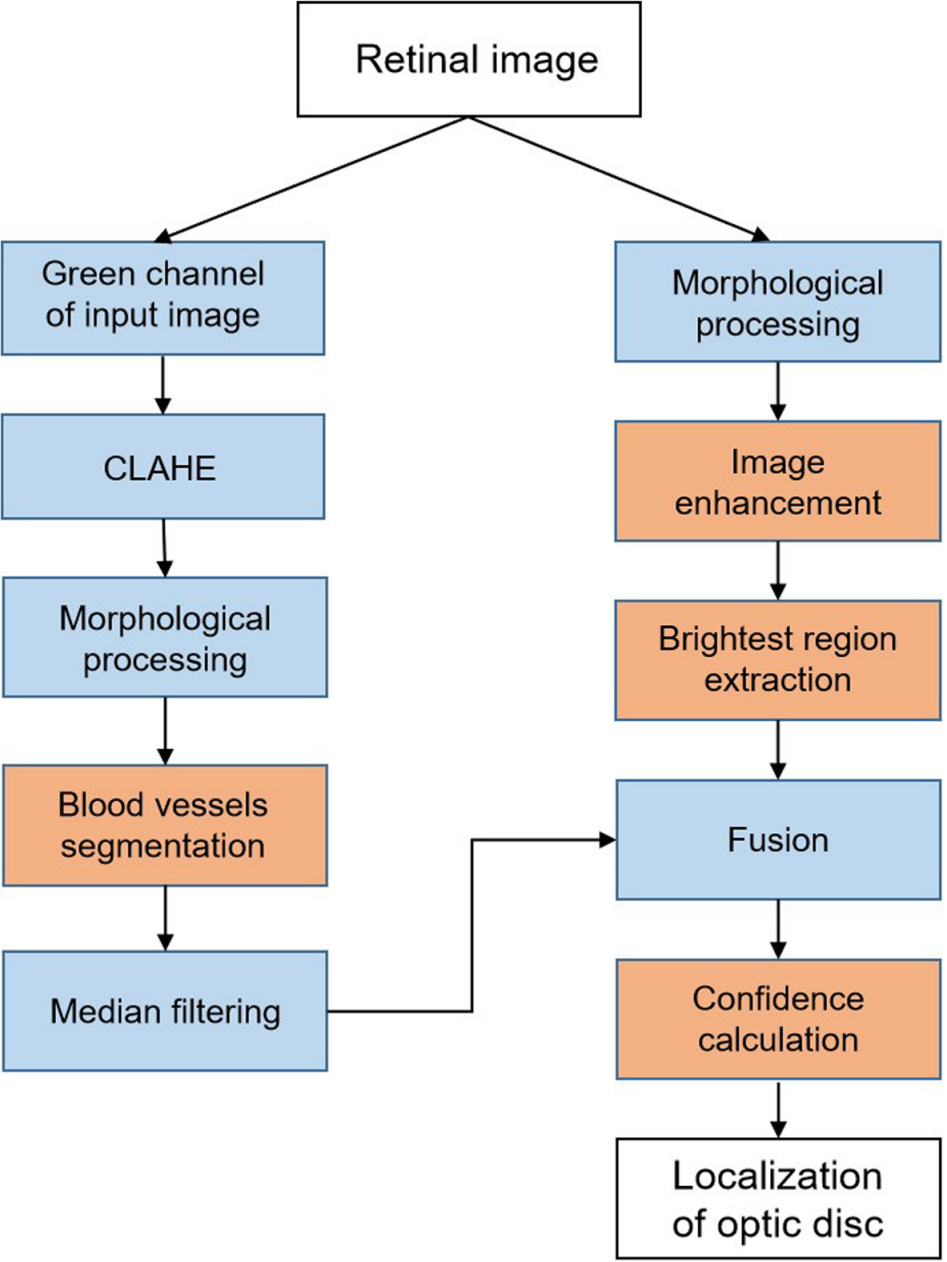
Our OD localization algorithm flowchart The intermediate steps are shown as blue blocks and the key steps are shown as orange blocks.

The retinal image comprises the small portions of the OD and OC, which are tough to segment. To deal with this difficulty, an OD localisation algorithm is put forward, which integrates the intensity and the vessels, aiming at the localization of OD center of via the sliding window way. The sub-image obtained from the OD center is deemed to be the ROI region. Therefore, the segmentation of OD and OC is to be conducted on the cropped ROI. Figure 3 portrays the flowchart abided by the OD localisation algorithm. Three key steps can be seen to localize the optic disc: Image promotion and brightest area extraction, blood vessel extraction, as well as the sliding window’s confidence calculation. Going forward, the three steps will be discussed at length.

##### Step 1: image promotion and brightest area extraction

**Fig. 4:**
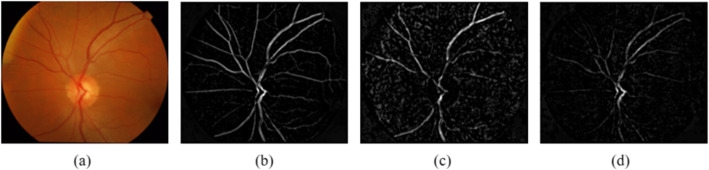
The vessel extraction result in different channel. **a** Input retinal image. **b** Extracted blood vessels in green channel. **c** Extracted blood vessels in red channel. **d** Extracted blood vessels in blue channel.

As a result of the various imaging conditions, morphological processing is conducted on the input image [Fig. 4(a)] to promote the retinal image as well as extracting brightest pixels out of the fundus. Top-hat transformation (*G*_*T*_) is employed to upgrade the interesting bright objects against the dark settings; bottom-hat (*G*_*B*_) upgrades the interesting dark object amidst a bright backdrop. Thereby, the upgraded gray-level retinal image (*F*') is explained as the follows:

1 $$ F\hbox{'}=F+{G}_T-{G}_B $$

Then, as the OD takes up brightest area of the retinal image, the pixels bigger than 6.5%of the maximum pixel value are deemed as the candidate pixels of OD.

##### Step 2: blood vessel extraction

In terms of the blood vessel extraction, Contrast Limited Adaptive Histogram Equalization (CLAHE) is used to upgrade the blood vessel within the green channel through the input image, for the reason that the blood vessels and the background in green channel contribute a better contrast than other channels. Next, the bottom-top hat transformation is made for the blood vessels extraction. Considering that the blood vessels is inclined to harbor smaller intensity than those of background, the blood vessels can be put extraction form the differences of bottom-hat transformation from the top-hat transformation. Additionally, median filtering is conducted to cut the salt and pepper noise of the blood vessel segmentation result. The process is expressed as follows

2 $$ {F}_{vessel}={G}_B-{G}_T $$

The vessel extraction result in green, red and blue channel can be obtained, as Fig. 4(b) Fig. 4(c) and Fig. 4(d) demonstrates**.** It confirms that the green channel contributes best effect of blood vessel extraction.

##### Step 3: confidence calculation for the sliding window

To realize effective localization of the OD, sliding window is conducted for scanning three different feature maps, which include brightest region of gray-level retinal image, blood vessels, and the fusion image combining brightest area with blood vessels. Have *f*(*i*), *f*(*bv*) and *f* (*ibv*) stand for the scores of each sliding window scanned via the three feature maps: intensity map *I*, blood vessel map *bv*, as well as intensity & blood vessel map *ibv*. Additionally, min-max normalization is used for the scores of sliding windows within each feature map, aiming to standardize the data between 0 and 1. Accordingly, the final scores of each window *S* refers to the mean value owned by *f(i)*, *f (bv)* and *f (ibv)*. At last, the localization of the sliding window with the maximum score is to be deemed as the location of OD.

**Fig. 5:**
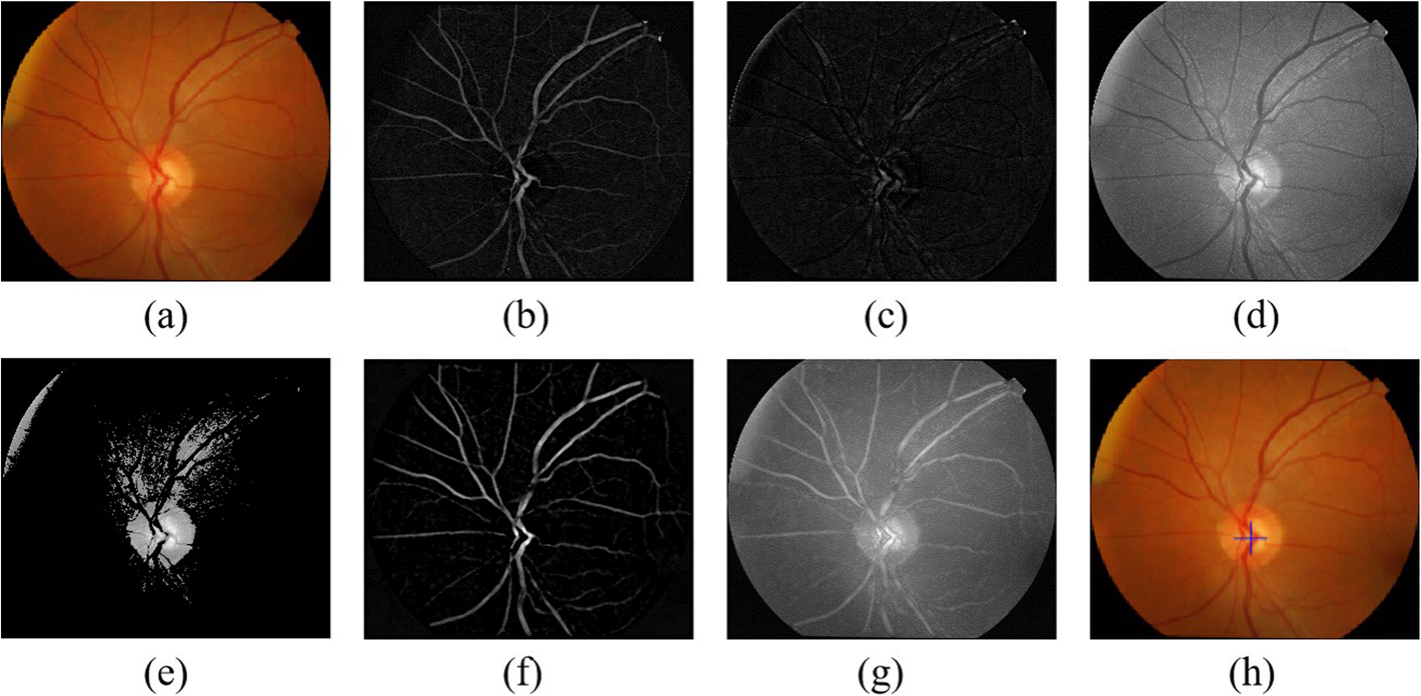
Key steps for OD localization. **a** Input retinal image. **b** Bottom-hat transformation result. **c** Top-hat transformation result. **d** Enhanced retinal image by bottom-top-hat transformation. **e** Brightest region of retinal image. **f** Extracted blood vessels in green channel. **i** Fusion image which combined enhanced retinal image with the blood vessels. **j** Our OD localization result

Figure 5 shows the key steps for OD localization. Bottom-hat and top-hat transformation have the result, which can be seen in Fig. 5(b) and Fig. 5(c). Figure 5(d) reveals the result of the enhanced retinal image from bottom-top-hat (BTH) transformation, the OD area is evidently upgraded; the gray-level retinal image also harbors an enhanced contrast. As shown in Fig. 4(e)**,** the brightest area of the retinal image is extracted, then the vessel extraction result *F*_*vesse*l_ are achieved as Fig. 5(f) **shows**. Based on the fusion image [see Fig. 5(g)], the location of OD [see Fig. 5(h)] can be obtained by confidence calculation for the sliding window.

**Fig. 6:**
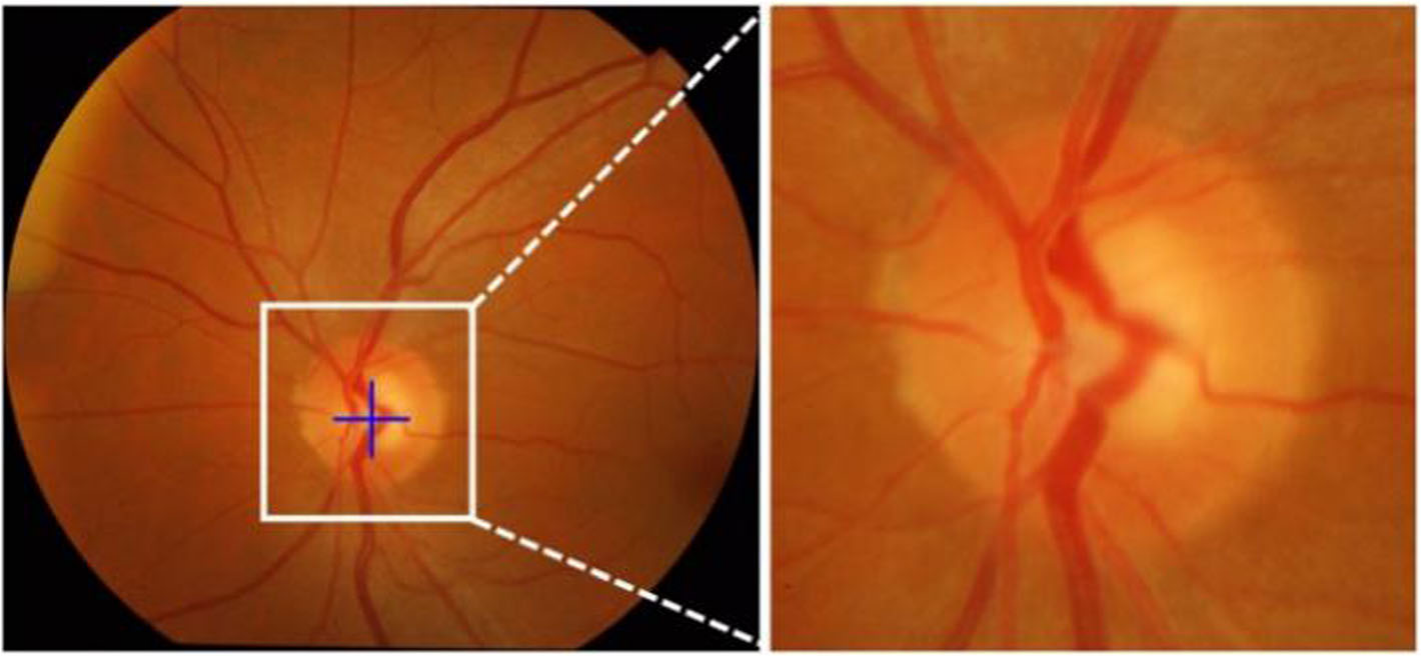
OD localization result and cropped ROI region

When the OD location is made, the square area which contains OD is put under extraction from the retinal image as ROI. Our work demonstrates that all the ROI areas take the same size, which is 1.5 times of the maximum diameter of OD. Where the calculation of the maximum diameter of OD is made by the OD mask of retinal images based on the existing dataset previous to OD localization. According to the experiment on test images, our approach can extract the OD inside the ROI area in an effective way. Figure 6 offers an illustrative example.

**Fig. 7:**
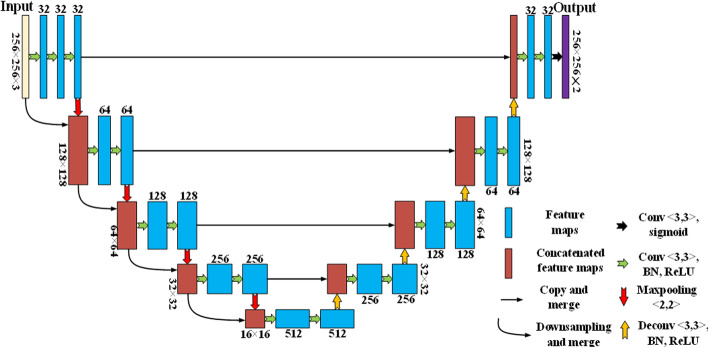
Our proposed network architecture

When the OD location occurs, the segmentation of OD and OC is achieved by our newly suggested network, which is called U-Net + CP + FL. It is made up of U-shape convolutional architecture (U-Net) [16], connecting path (CP) with fusion loss (FL) function, as Fig. 7**shows**. According to Figure, the network is composed of three components: (i) an architecture of U-shape improved network (ii) concatenating path — a supplemented connection design between encoder layers, and (iii) multi-label output layer in connection with fusion loss function.

### U-shape network architecture

U-shape network refers to an effectively powerful and fully convolutional neural network oriented with biomedical image segmentation even built for small dataset. The network is made up of two parts: encoder path, decoder path, and skip connections. Encoder path shoulders the responsibility of feature extraction. It is made up of convolutional block, which includes batch normalization (BN), ReLU activation and convolutions in a row. Maxpooling is applied to reducing the resolution to the feature maps. Decoder path works as a reverse process of the encoder path, trained for the construction of the input image resolution. In order to restore the resolution to the feature maps, deconvolution is utilized in the decoder layer, thus matching pooling layer in the encoder path. Eventually, the output at the final decoder layer is used to satisfy a multi-label classifier. Skip connection functions as a crucial design in encoder-decoder networks. The skip architecture undertakes to relay the intermittent feature maps, following the route of encoder layer to the matched decoder layer. That not merely contributes to the reconstruction to the input image resolution, but solves the vanishing gradient problem.

### Concatenating path

Given the inspiration from Densenet [17], new connections are introduced between encoder layers --concatenating path. It gives rise to the feature maps sharing and multi-scale inputs oriented with the encoder path. On the concatenating path, the input of current layer is made up of last pooling output and last resized input. Thereby, not only does the encoder path receive feature map from the last layer, but also it achieves the input layer and the semantic information based on all the previous layers. That is equal to multi-scale inputs and featured by maps sharing. Experimental results are demonstrating that our suggested network helps enhance the segmentation accuracy.

### Multi-label loss function

OD and OC account for small sections of retinal image; therefore, overfitting tends to occur and even prone to trained on the cropped ROI area. According to U-NET + CP + FL, the proposal is to combine the weighted binary cross-entropy loss and the dice coefficient loss as the object function for optimization, where the introduction of dice coefficient can enliven again the data imbalance problem. As for the proposed network, the output result is comprised of two channels consistent with OD and OC segmentation mask separately. Thereby, multi-label loss is signaling that the pixel should independently belong to OD or/and OC, which lessens the data imbalance as well. The multi-label loss function is interpreted as follows:

3 $$ L={\lambda}_1\left({L}_{CE}^{disc}+{L}_{dice}^{disc}\right)+{\lambda}_2\left({L}_{CE}^{cup}+{L}_{dice}^{cup}\right) $$

4 $$ {L}_{CE}=-\sum \limits_{i=1}^N{q}^i\cdot \log {p}^i\kern0.5em ,\kern0.5em {L}_{dice}=-\sum \limits_{i=1}^N\frac{2\left|{p}^i\cdot {q}^i\right|}{{\left|{p}^i\right|}^2+{\left|{q}^i\right|}^2} $$

Where *L*^*disc*^_*CE*_ and *L*^*cup*^_*CE*_ individually stand for the cross-entropy loss of OD and OC; *L*^*disc*^_*dice*_ and *L*^*cup*^_*dice*_ refer to the dice coefficient loss. *p*^*i*^ means the predicted probability of pixel; *i* belongs to OD in OD segmentation mask or to OC in OC segmentation mask; *q*^*i*^ indicates the ground truth label for pixel *i*. λ_1_ and λ_2_ in Eq. (3) tells trade-off weights to determine the contribution of cross-entropy loss as well as dice coefficient loss. Our work designs λ_1_ and λ_2_ to 0.5.

### Training details

According to our work, the segmentation network is constructed with Keras, a high-level neural networks framework. The network is under the training based on backpropagation algorithm. The ORIGA [18] retinal image dataset is used for the training and evaluation of our proposed network; 450 retinal images are chosen in random for the training, 50 for the validation and the 150 retinal images left for the test. The training data make for the cropped ROI areas containing the optic disc area as well as the relevant cropped optic disc as well as optic cup segmentation masks. In the duration of training, ADAM method is employed for the network optimization based on initial learning rate of 0.0001. 256 × 256 is used for all the resized training images as well as masks. In addition, such factors as random flip and rotation, color jitter and random affine transformation are applied to training images augmentation; the training of the network is set with 500 epochs.

**Fig. 8:**
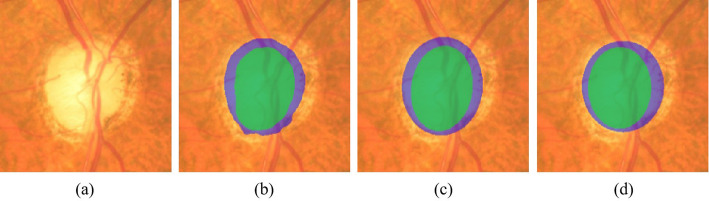
CDR calculation result. **a** Original fundus ROI region. **b** Our network segment result. **c** Our ellipse fitting result. **d** Ground truth

**Post processing** Fig. 8(b) includes an illustrative instance for the simultaneous segmentation of OD and OC. In order to attain a precise cup-to-disc ratio (CDR) measurement, it can mitigate the effects which and uneven boundaries exert to post-process the segmentation result. Most isolated points will become obsolete through erosion and dilation operations. As OD harbors another distinct feature -elliptical shape, we choose the least-squares optimization to suit an ellipse with the segmented OD contour, where the extraction of the contour pixels is made in virtue of a canny edge detector. In the end, the centroid and the long/short-axis length of the OD, achieved by ellipse fitting are conducted to cover ellipse on the input image for segmenting OD of the input retinal image. The identical operations are made on the OC. Figure 8(c) reveals an instance of ellipse fitting result. It can be easily seen that one can both OD and OC boundaries are of more smoothness and closeness to the ground truth following performing ellipse fitting, as Fig. 8(d) shows.

#### Measurement of clinical parameters and Glaucoma risk prediction

When OD and OC are segmented at the same time, some key clinical parameters, like CDR-relevant parameters as well as the ISNT-related parameters could be achieved for glaucoma screening. In terms of the CDR-related parameters, the calculation of vertical CDR is made through the rate of the vertical OC diameter (VCD) for the vertical OD diameter (VDD). Other CDR-related parameters are made up of horizontal CDR, optic disc area, optic cup area and CD area ratio, and so on.

**Fig. 9:**
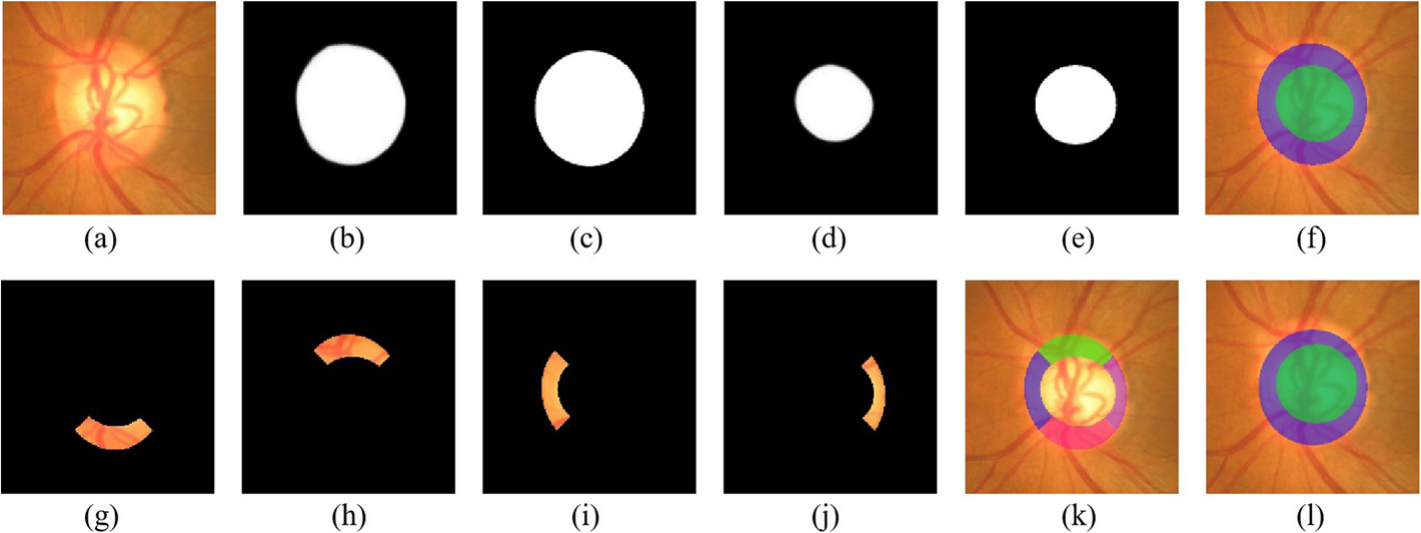
OD and OC segmentation result and the cropped NNR corresponding to I, S, N and T regions. First row illustrates the OD and OC segmentation result of fundus ROI region. **a** Original fundus ROI region. **b** OD segmentation of our method. **c** OD segmentation of ground truth. **d** OC segmentation of our method. **e** OD segmentation of ground truth. **f** OD and OC ellipse fitting result of our method. Second row shows the cropped NNR corresponding to I, S, N and T regions. **g** INR. **h** SNR. **i** NNR. **j** TNR. **k** Visualization of NNR on ROI. **l** OD and OC ellipse fitting result of ground truth

As for the ISNT-related parameters, neuro-retinal rim (NRR) is dissected into four different areas, which are respectively superior NRR region (SNR), inferior NRR region (INR), nasal NRR region (NNR) as well as temporal NRR region (TNR), in accordance with the distribution of the inferior (I), superior (S), nasal (N), temporal (T) regions in ISNT rule, as shown in Fig. 9(g) to Fig. 9(j). Other ISNT-related parameters harbor the ingredients: INR to disc area ratio, thickness of INR area and INR area. Moreover, the extraction of INR to OD area ratio and thickness of INR area can be achieved as clinical parameters, which helps the glaucoma model to judge whether or not the NRR is abiding by the ISNT rule. To sum up, the extraction of 25 features correlated with segmented OD, OC and NRR analysis are conducted totally. Figure 9 demonstrates an interpretation instance segmented OD, OC and NRR areas.

Aiming to prevent noisy, redundant or unimportant characters to be brought into the train classifier, it is suggested that an optimal feature set chosen. Accordingly, three different feature selection approaches are taken in our job for the removal of the redundant characteristics.

**Fig. 10:**
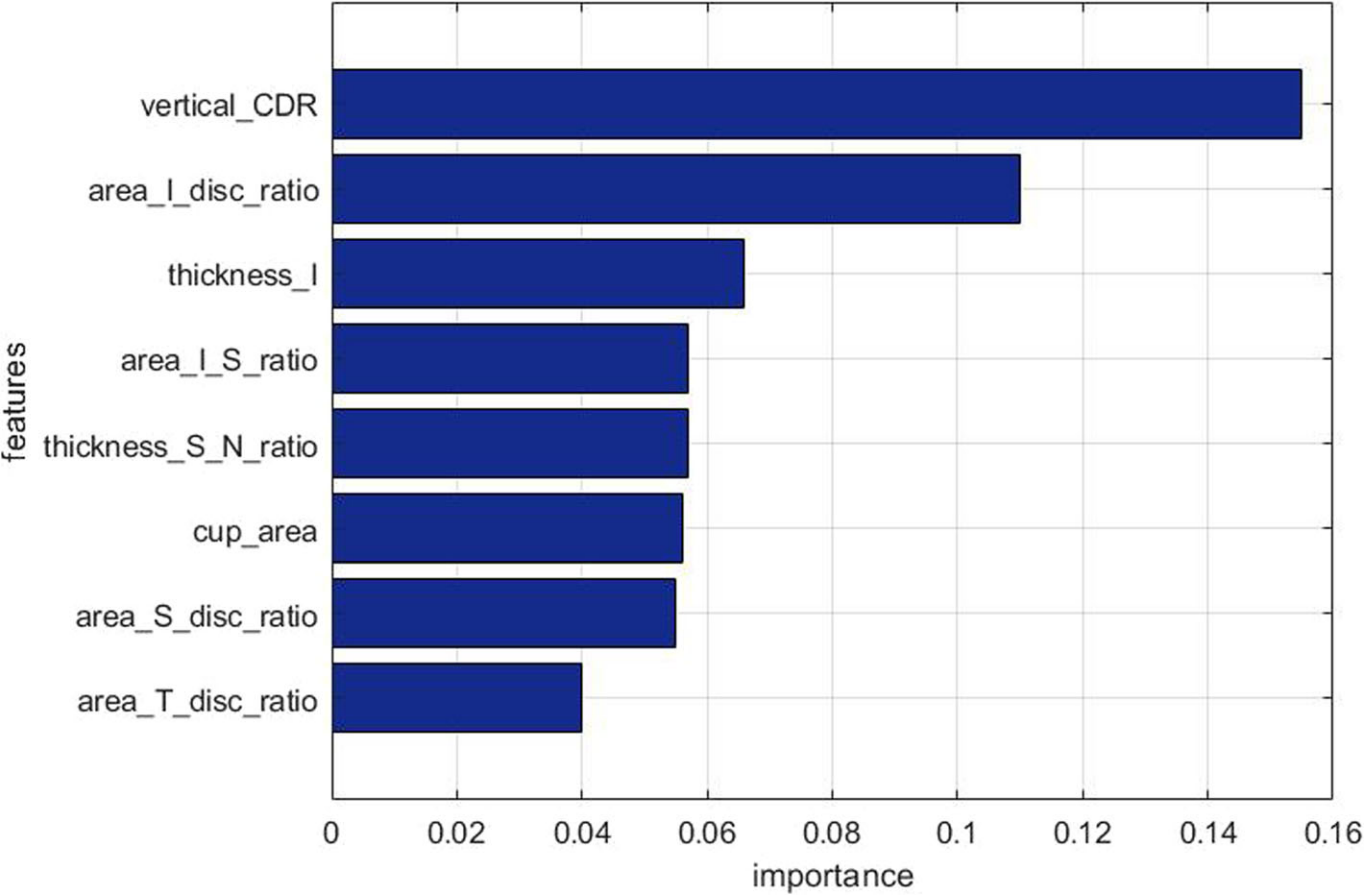
Bar graph of the Feature ranking of the selected features. More details about the selected features please see Abbreviations

In particular, variance analysis is applied to finding the low-variance-based, indicating their constantly or approximately distributed near the same value. Next, the correlation analysis is employed for the measurement of the correlation between two variables with strong correlation represent redundant features. These relevant features are contributing less or least to glaucoma classification; or even misleading the classifier. Then, feature ranking is used for identifying and ranking the significant characteristics, which are of most relevance to glaucoma classification. Features are here ranked in virtue of the random forest model. Gini index is used for the measurement of dataset purity. Figure 10 displays the final feature importance rank, in combination with 10 decision trees for the random forest model. On the basis of the statistic result, the eight features, with the most significance relevant to glaucoma classification are chosen for the training of the glaucoma classifier.

Attention should be paid to it that imbalanced data gave rise to a dramatic decay in accuracy performance of the classifiers, for the reason that their being biased to the majority class [19]. Therefore, according to our work, synthetic minority over-sampling technique (SMOTE) sampling [20] and class weight approach are utilized for the balanced number of features within the normal and abnormal classes. Our assignment means that glaucoma screening belongs to a binary classification problem; thus, three candidate classifiers, support vector machine (SVM), random forest (RF) or gradient boosting decision tree (GDBT) can be employed for glaucoma classification. Nevertheless, RF classifier cannot function well in the circumstances of the random sampling as well as inability to data projection. They harbor no suitability for glaucoma detection. Additionally, CDBT, which is able to attain higher accuracy than SVM can also harvest stable robustness. Therefore, different from our previous work [21], the application of GDBT classifier adopted in this work, is aimed at the automated characterization of the glaucoma and non-glaucoma classes. 10-fold cross-validation is employed for the evaluation of classification performance.

### Mobile app detail

**Fig. 11:**
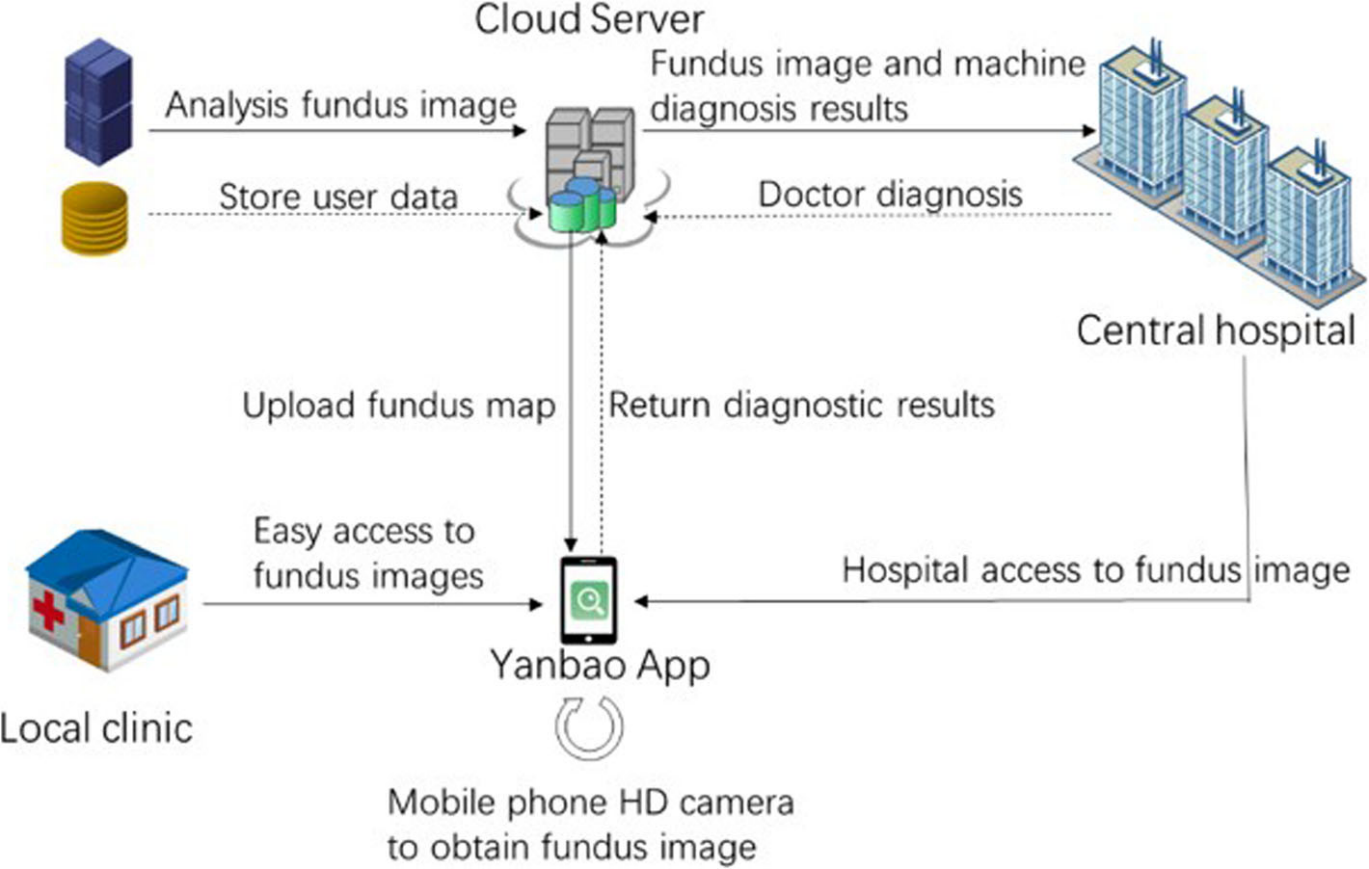
Overview of our App application

Figure 11 interprets the App application’s functionality as well as operational program. Thereby, through the professional retinal cameras doctors adopts, users are able to achieve their high-quality retinal images by hospitals and clinics. Besides, the fundus images can also be achieved in virtue of the oDocs Fundus [15]. The App, which could be used for uploading the fundus images, will be given server-based glaucoma lesions feedback. During the shortage of medical resources and the increasingly patients’ demand, this App will powerfully mitigate the contradiction through its aided diagnosis.

#### User Interface

**Fig. 12:**
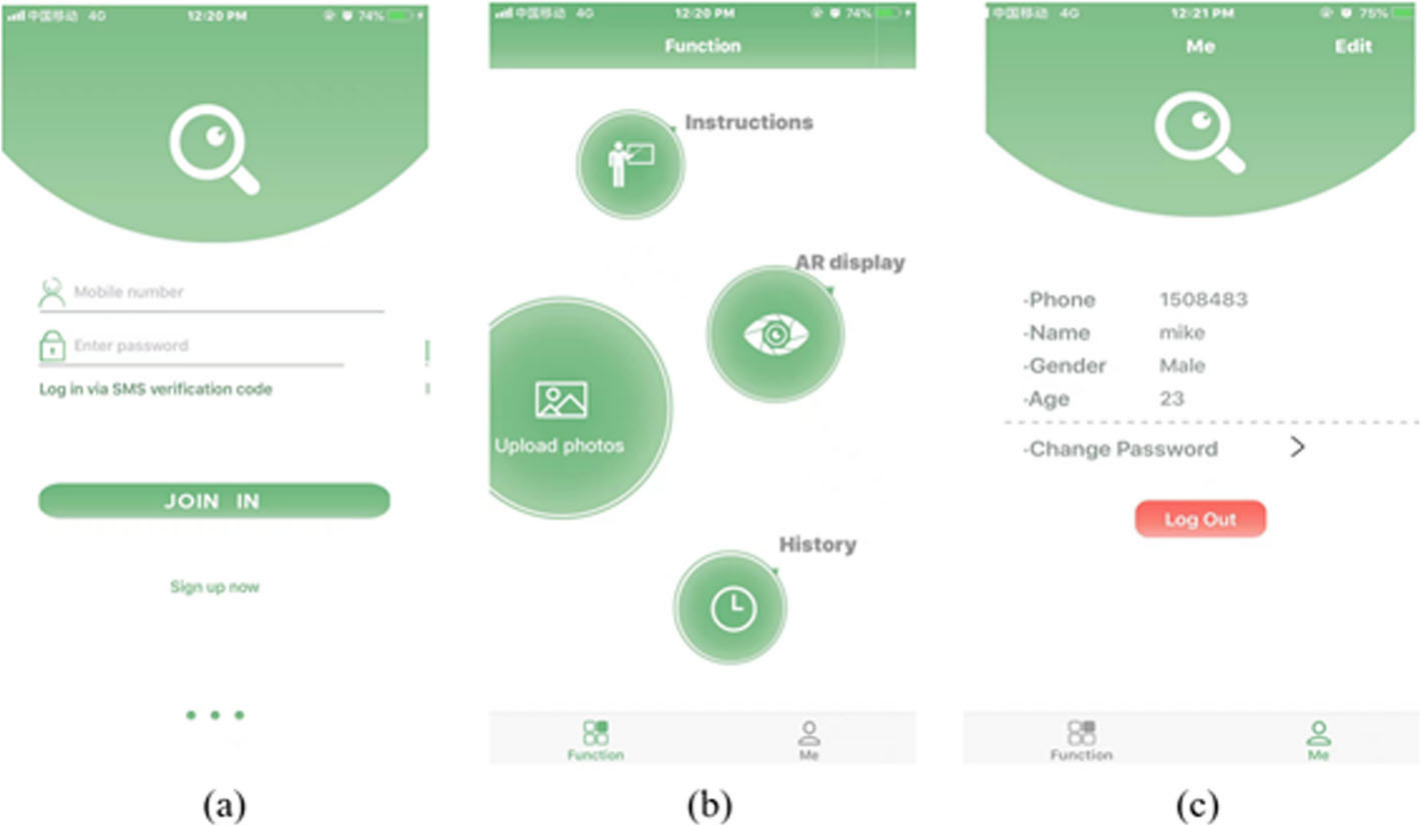
Login interface and home interface

Our target is to scheme an easily intuitive App, so a user-friendly interface is indispensable. Figure 12(a) shows the login interface of the App. The overall design is neat and practical. Figure 12(b) presents the home interface of the App. There are four main functions in home interface, including instructions, uploading images, AR display and history. The design of the homepage clearly shows the core functions and features of the product, users can easily find the module they need. In addition, users can modify their personal information and log out in “Me” interface as shown in Fig. 12(c).

**Fig. 13:**
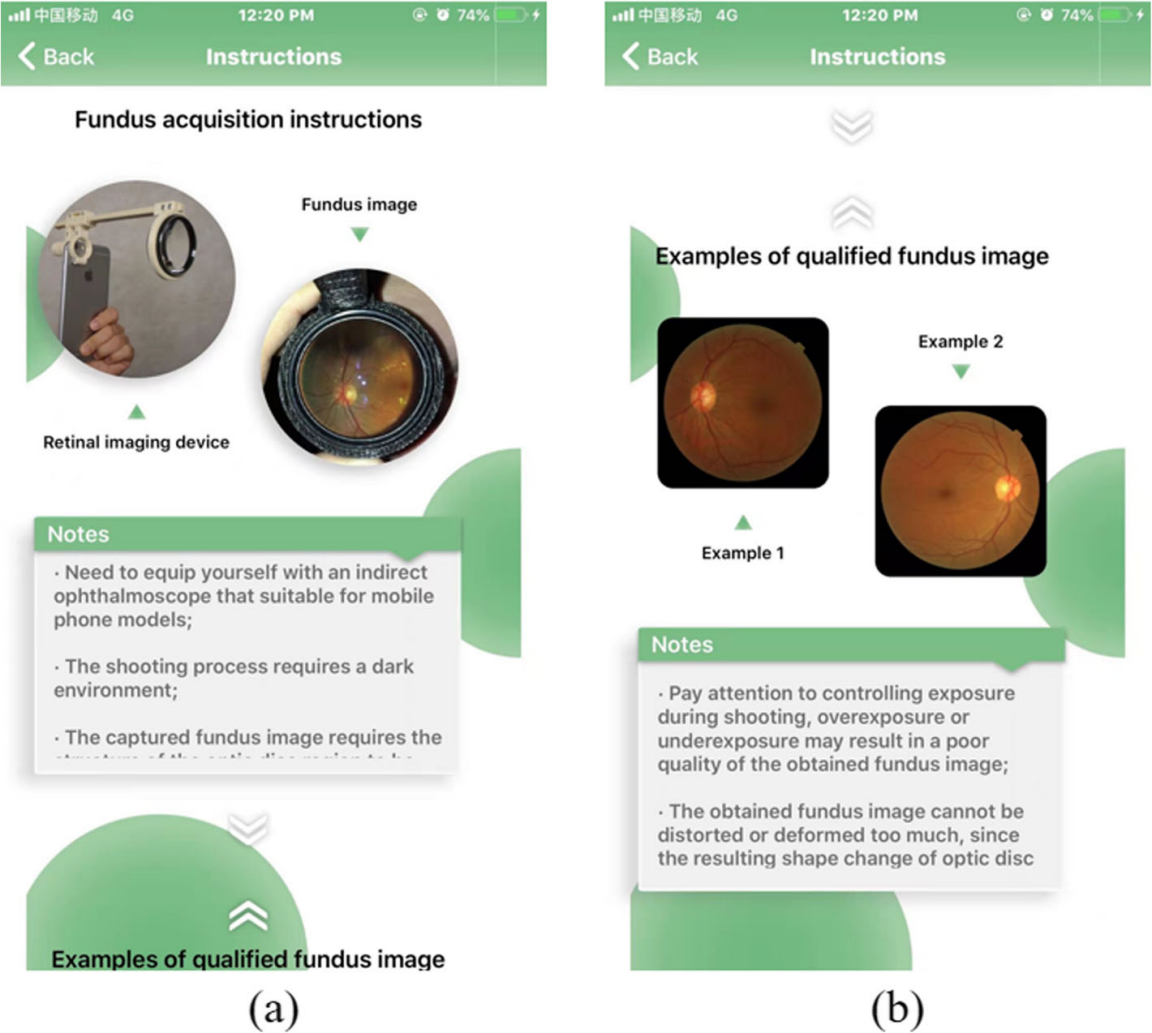
Instructions interface

A straightforward instructions interface is very helpful for users who are not familiar with this App. Figure 13 shows the instructions interface of the App, fundus acquisition instructions and example of qualified fundus image are presented in Fig. 13(a) and Fig. 13(b)**.**

**Fig. 14:**
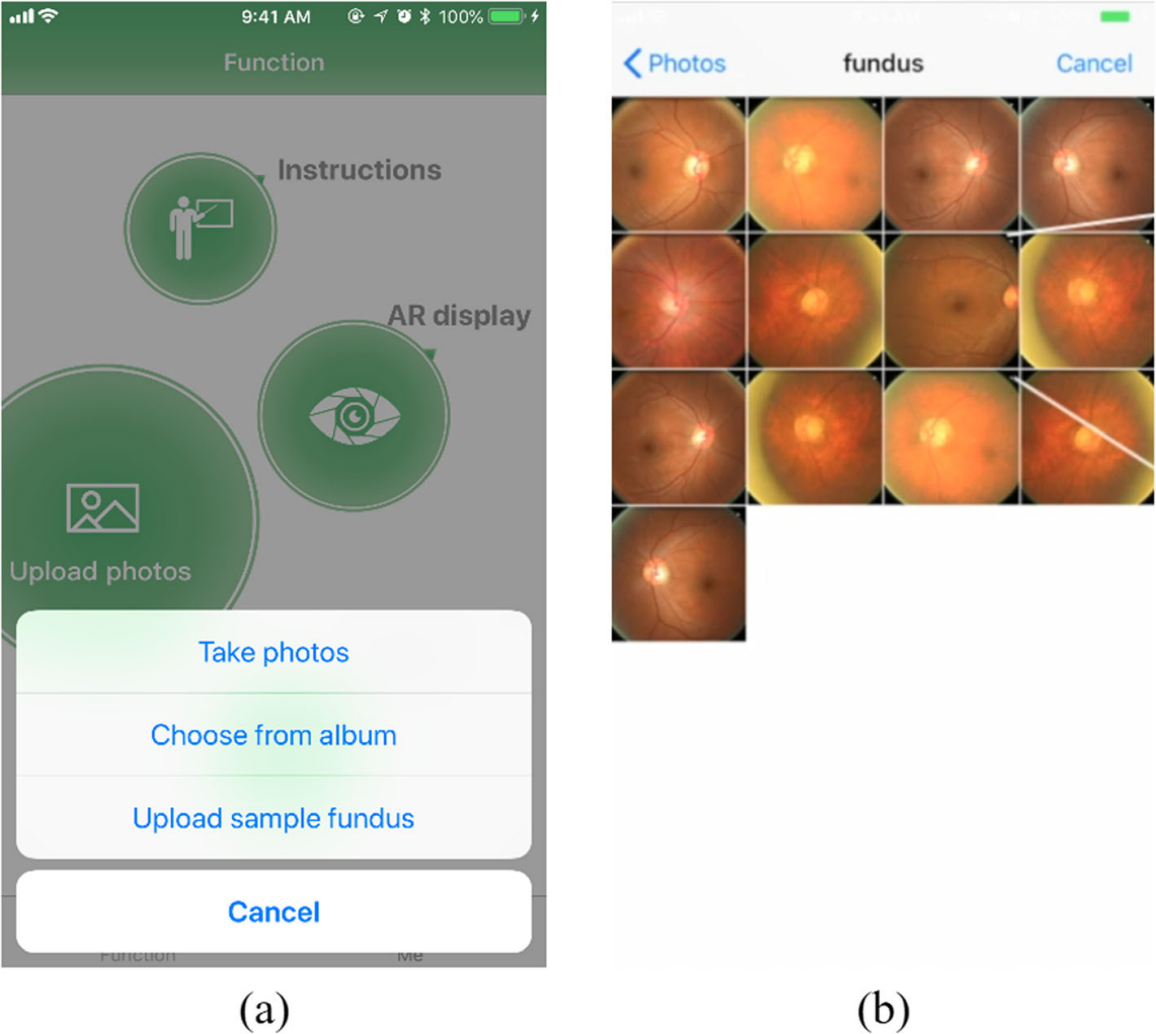
Interface of uploading photos for the home screen

Figure 14 symbolizes the App’s interface for photo loading. The App specifies that the users can upload personal retinal images under two patterns. Here is the specified operation: click “upload photos” on the home screen, which is called mode selecting for fundus image uploading. In the duration of the process, it requires taking the oDocs Fundus to obtain the fundus image in virtue of the mobile phone’s high-definition camera [15]. Moreover, the approach to achieving and uploading the retinal images in virtue of the album, the users are allowed to employ the relatively high-quality fundus images the ophthalmologist have taken in the hospital. After selecting uploading the fundus image, what the system will do is to automatically have the fundus image uploaded in virtue of taking the photo album image as the user have chosen to do.

**Fig. 15:**
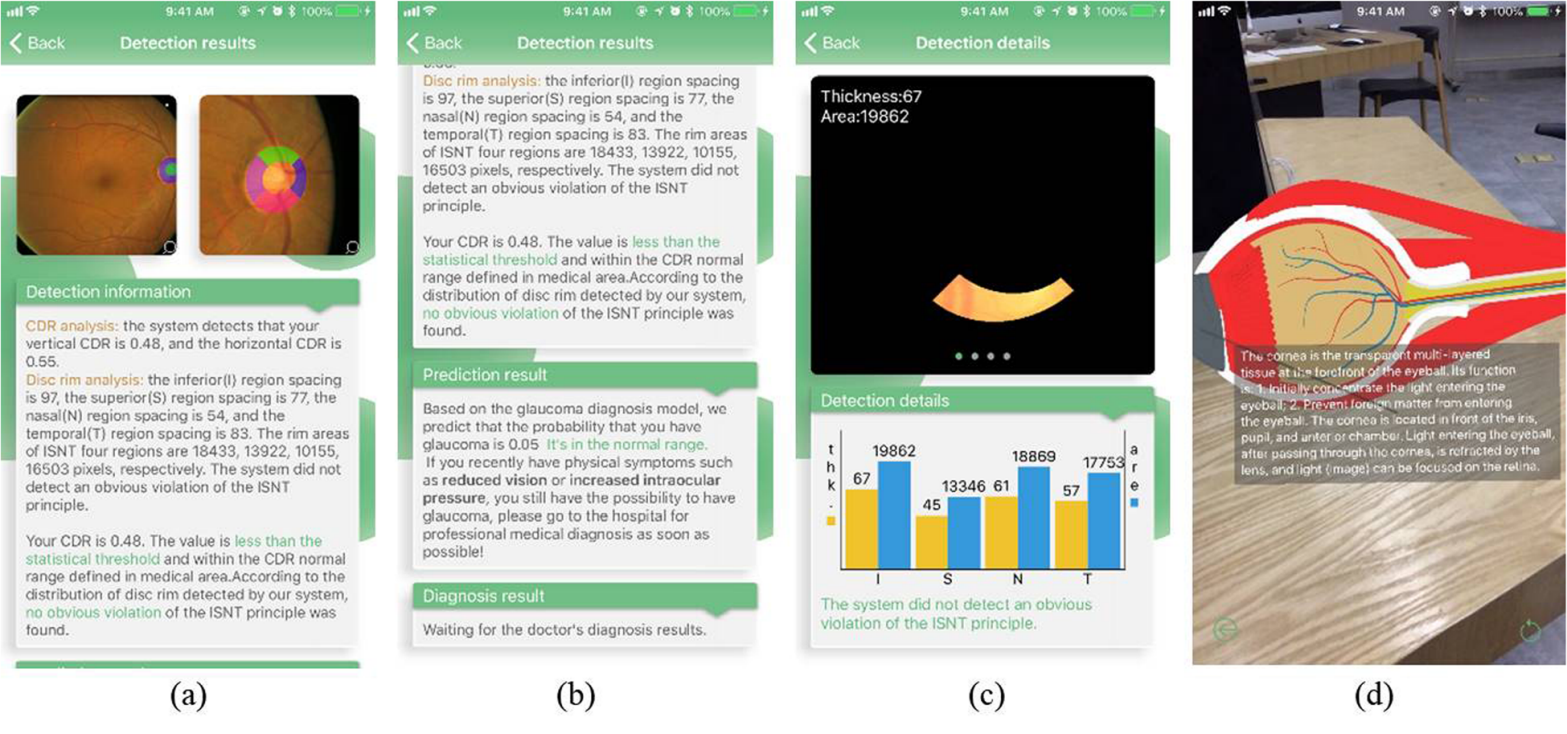
Examples of detection feedback interfaces

The feedback achieved from this App after the upload of the fundus image is composed of four parts: CDR analysis, NNR analysis, glaucoma classification, as well as doctor’s diagnosis display. The feedback of the first three can be made in 10 s; the doctor’s diagnosis requires to be explained by a professional medical work or a doctor. As soon as the diagnostic results are achieved, the users will read the results through sliding up and down, or on image of the image segmentation on the diagnostic result interface in order to examine the detailed information about the optic cup as well as disk image. Figure 15 tells some instances of detection feedback interface. As the Figure displays, the clinical parameters correlated to CDR and NNR analysis are demonstrated in Fig. 15(a). These parameters contain the location of the OC and OD, vertical as well as horizontal CDR, the specific distribution of inferior (I), superior (S), nasal (N) and temporal (T) areas for NNR, etc. additionally, the NNR analysis on the basis of the area, thickness and area rate among them are committed to validate the ISNT rule, as Fig. 15(c) **shows.** According to Fig. 15(b) Glaucoma risk prediction can be conducted **in light of** these detected index values considering that the design of our App aims to serve doctors as their aided instrument, therefore, doctor’s diagnosis display is also offered by the App [Fig. 15(b)]. Furthermore, for ophthalmology popularization, the AR effect interpretation on the eyeball structure is given in our App as well, as Fig. 15(d) explains. That means that users can click the button “AR display” on the home screen to enter AR display interface. Thus, they can have a better comprehension of their own eyes, thus achieving the self-protection aim with self-awareness.

**Fig. 16:**
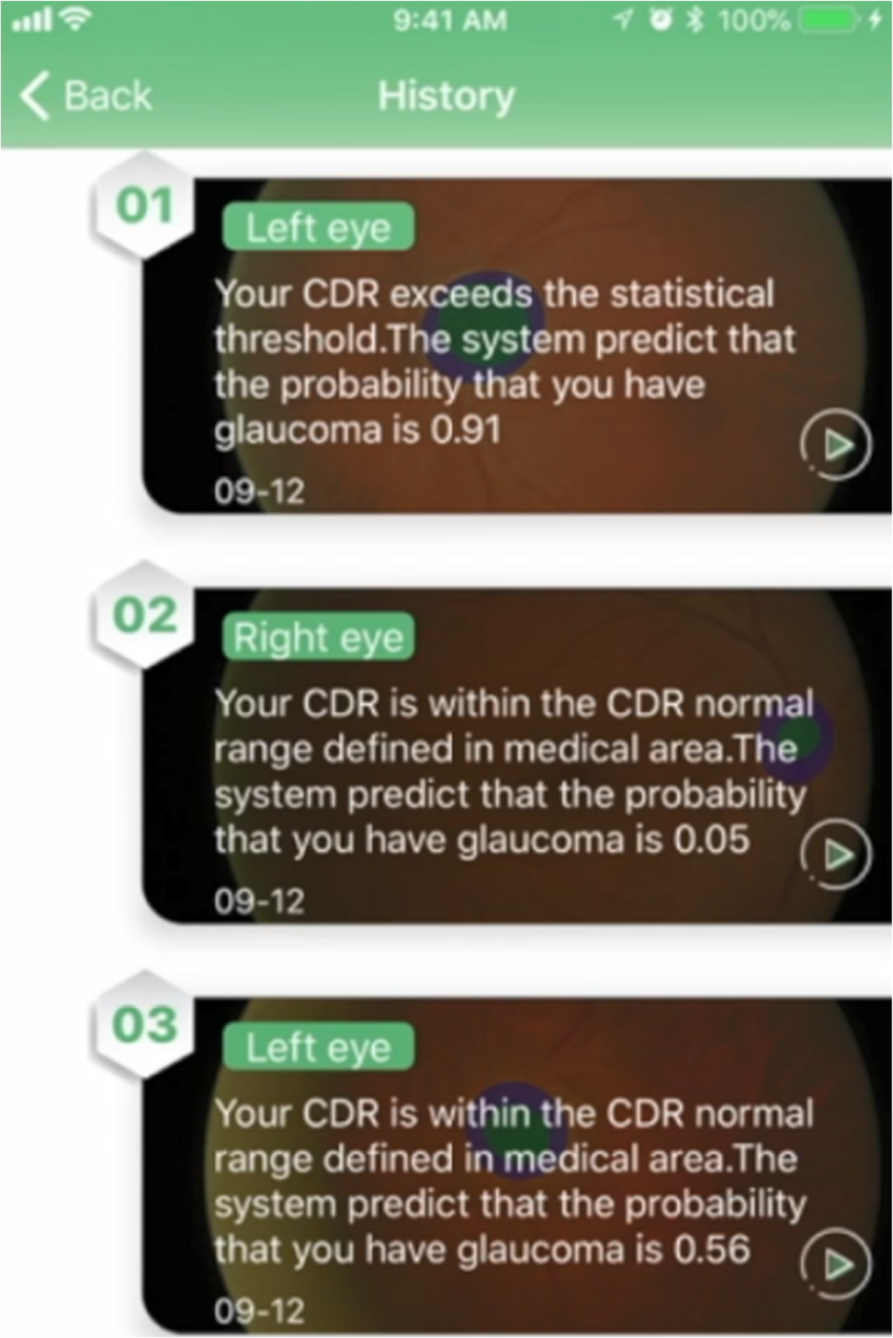
Interface of detection history

Figure 16 presents the App’s interface of detection history. The results of detection are recorded, and users can click the “History” button on the home page to view the history, keeping an eye out for the changes in their own condition.

To sum up, the interface of the APP is very neat and easy to understand, the layout of the page is exquisite. Moreover, the green style of the App can help relieve eye strain for users. Considering that glaucoma is most common in middle-aged and elderly people and some glaucoma patients may suffer from impaired vision, Yanbao App offers a user-friendly UI for them.

#### Implementation method

**Fig. 17:**
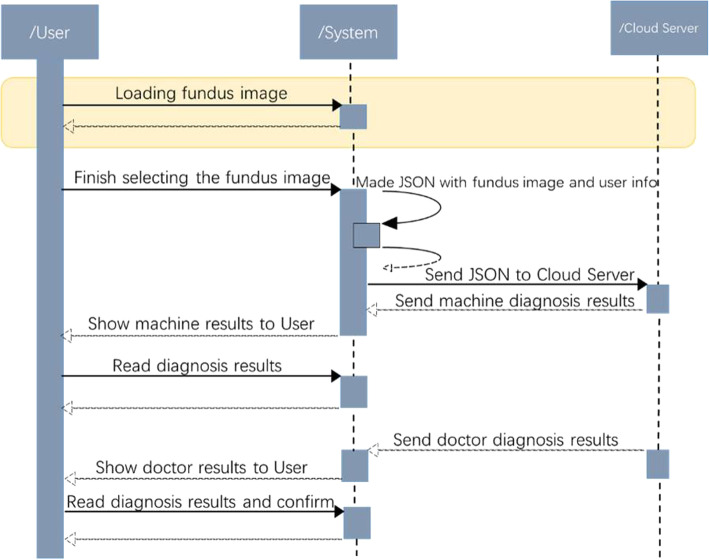
Sequence diagram of the diagnosis process

Figure 17 offers the sequence diagram illustrating the diagnosis process. According to the sequence diagram, the users may select a mode for loading the eye fundus image. During the step, the users need to choose their target fundus image. To achieve this, the system will make the first decision on whether or not the image what the users have uploaded is a fundus image. Provided that it is a non-fundus image in the range of a natural image, a hint will be provided that it is a non-fundus image considering the image analysis is invalid. Moreover, while conducting the fundus image, work should be done to avoid the underexposure or overexposure. The distortion of the visual disk shall be reserved in a small scale as could as possible. The main reason is that the image’s exposure and distortion will exert a negative influence on the final analysis effect.

After determining the fundus image, the system will receive and then have the fundus image and the user information converted into JavaScript Object Notation (JSON) file. Next, JSON file will be transferred to the cloud server. The Python script kept in the cloud server undertakes to receive and process the JSON file. JSON is the file format where the system makes communication with the cloud server. The results, which are achieved after the process, are to be sent back and then put into storage in the database attached to the database management system (DBMS). As such, our App diagnostic information is to be signaled to the doctor. The diagnosis results are to be signaled to the cloud server following the doctor’s diagnosis. Finally, the server will have the final diagnostic information sent to the users.

Yanbao App requests Web server as the information sender through Web, the POST request in particular. The request is made up of the JSON file and the URL attached to the cloud server managing information with the acceptance of script. When receiving a request, the Python script will conduct different operations on JSON files in accordance with different URLs, and then upadating, adding or deleting the database. In accordance with the fundus image in JSON, the moment the request is sent for detecting the fundus image, the Python file will conduct process and analysis on the image, have the detection result returned to the mobile facility by taking the form another JSON file. With this method, the users may conduct detection and updating on fundus images.

**Fig. 18:**
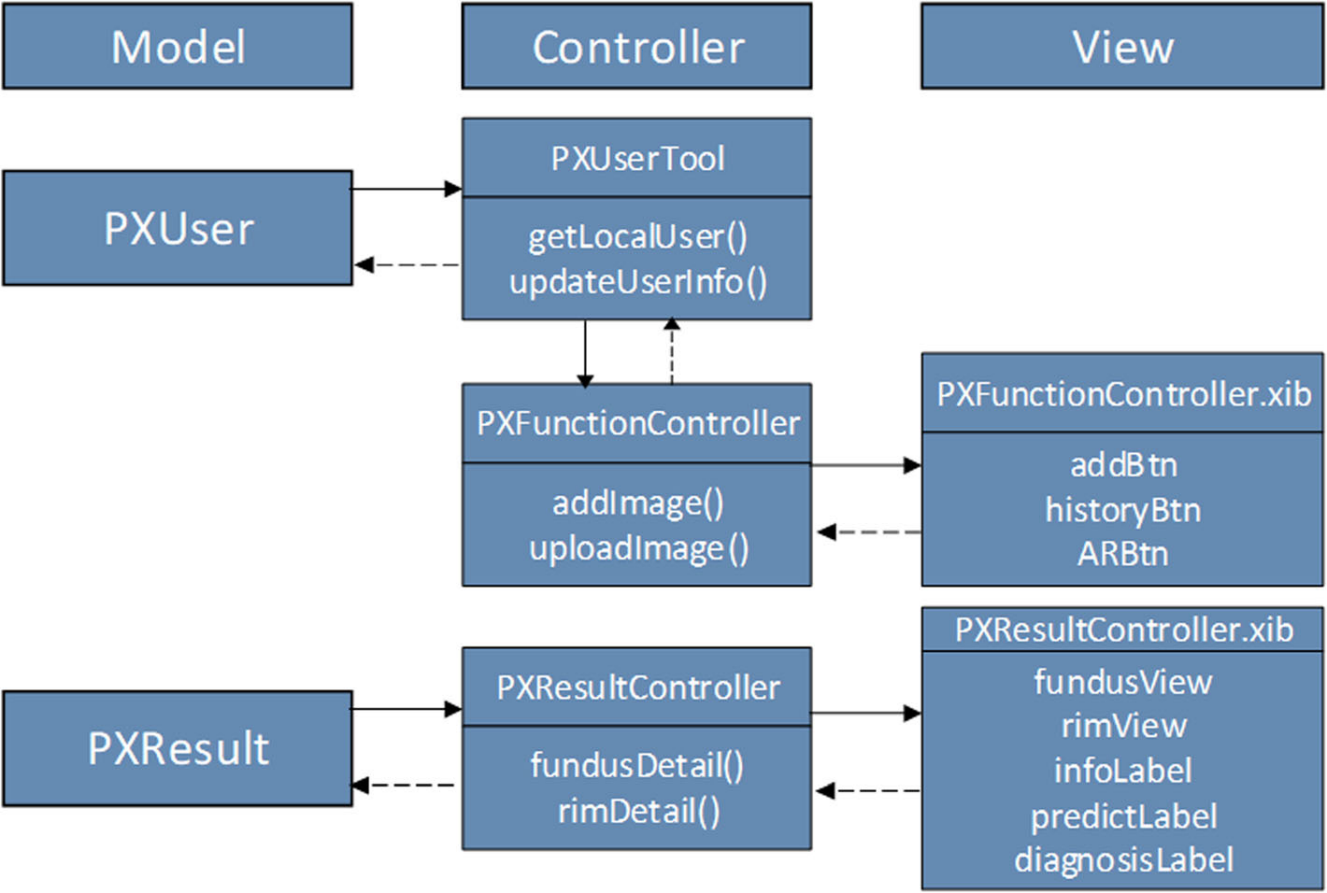
Class diagram excerpt of Yanbao app

Yanbao App is constructed under the major frame, which is displayed in Fig. 18’s class diagram structure. The whole software follows the design in the architecture pattern of model-view-controller (MVC). The architecture model abides by the data storage; the view is for the interpretation and the controller is oriented with data processing, linking views and models. While making detection-oriented fundus image uploading, the “PXUserTool” class manages to catch the local user information (e.g. user ID) modeled after the “PXUser” class sending it to “PXFunctionController” class. Furthermore, the “PXFunctionController” can load the fundus image as a response for the touch event, then convert the local user information to a JSON file to have it uploaded into the cloud server. More than that, the “PXFunctionContrller.xib” class undertakes to display the interface and get touch events. PXUserTool is put under singleton mode to guaranteed the merely one local user. Nevertheless, the interface file, in the same name as Controller and suffix xib, is supposed to be the Controller of the identical name, ensusuring that the events achieved on the user interface will received the response from the controller. Additionally, on the prerequisite of the returns of the detected JSON file, the “PXResultController” class manages to take the JSON file, convert the file into the “PXResult” model, anthen display the converted model onto the “PXResultController.xib” class.

The Yanbao App is technologically originated by the official development framework structured by iOS applications, whose advantages are of high fluency based on mobile phone carrier’s uniform specification. Also, the commonly furnished high definition camera is easy to be combined and the simple indirect ophthalmoscope to achieve fundus images. Additionally, the iOS operating system is a controlling operating system under the development of Apple Company of America perfectly designed and easily operated. The iOS application contains development framework and the XIB interface file, which are applied to completing the interface layout via drag-and-drop, and other approaches. All these functionalities showed relevance to achieve an intuition-based App. Our App is going on iPhone under the operation system iOS11 or the above; the App is of availability for downloading from Apple’s App store (http://url.cn/57tk9jT).

## Results

### Evaluation of proposed algorithm performance

To evaluate the performance of U-Net + CP + FL in segmenting OD and OC, quantitative assessment is performed on ORIGA datasets [18]. The balanced accuracy *Acc* and overlap score *S* are used to evaluate the segmentation performance. Here are the two indexes

5 $$ S=\frac{\mathrm{Area}\left( GT\cap SM\right)}{\mathrm{Area}\left( GT\cup SM\right)} $$

6 $$ Acc=\frac{\mathrm{Sensitivity}+\mathrm{Speciality}}{2} $$

In (5), *GT* and *SM* respectively demonstrate the ground truth as well as segmented mask. Area(.) means the region areas. The index *Acc* is made up of sensitivity (true positive ration) as well as specificity (false positive ratio). In terms of glaucoma diagnosis, CDR value performs a crucial clinical measurement. Thus, our CDR performance is put under evaluation with the absolute CDR error, which refers to *δ*_*CDR*_ = | *CDR*_*g*_ - *CDR*_*p*_ |. Now, *CDR*_*g*_ stands for the ground truth collected from the trained clinician; *CDR*_*p*_ represents the CDR out of the calculation of our proposed approach. Table 1 demonstrates the comparison results of U-Net + CP + FL and U-Net on ORIGA dataset.

**Table 1:** Segmentation performance comparison of different methods on ORIGA dataset

Method	*S*_*disc*_	*Acc*_*disc*_	*S*_*cup*_	*Acc*_*cup*_	*δ*_*CDR*_
U-Net	0.885	0.959	0.713	0.901	0.102
U-Net + CP + FL	**0.939**	**0.984**	**0.805**	**0.942**	**0.054**

Table 1 reveals that we are able to draw a conclusion that our proposed updated deep neural network U-Net + CP + FL obtains the better performance than U-Net on *S*_*disc*_, *Acc*_*disc*_, *S*_*cup*_, *Acc*_*cup*_*and δ*_*CDR*_, so it performs better than U-Net in segmenting OD and OC. The time complexity of the U-Net + CP + FL is 0.4160 s per image, and the image will be resized to 256 × 256 before being input to U-Net + CP + FL.

**Fig. 19:**
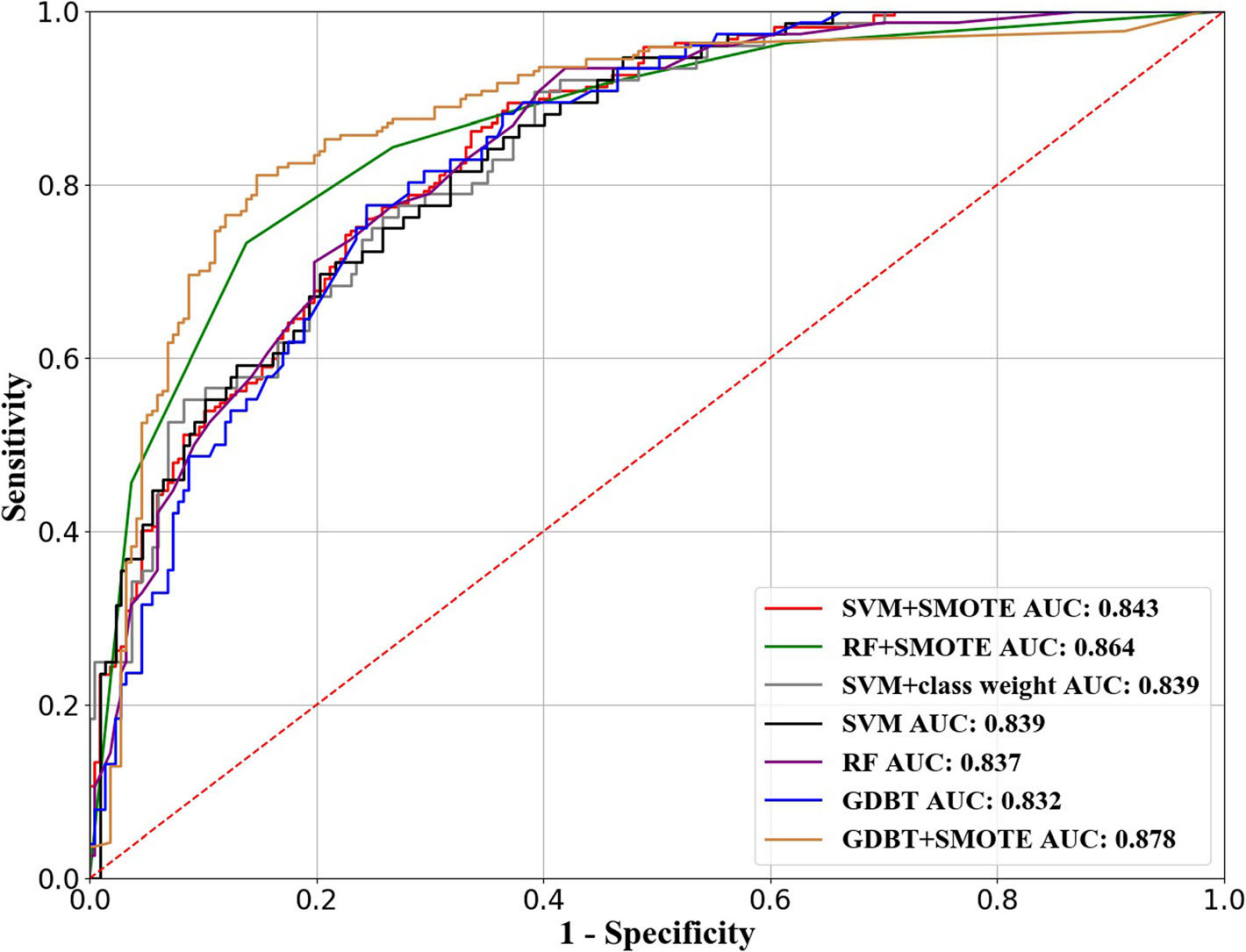
ROC curves with AUC scores of different methods for glaucoma classification

Quantitative evaluation is performed on ORIGA dataset [18]. Receiver Operating characteristic (ROC) [22] curve is used for the comparison of the performance of different glaucoma classifiers. More, four evaluation criteria are composed of: *Sensitivity* [22], *Specificity* [22], Accuracy (*ACC*) and area in ROC curve (*AUC*) [23] are conducted for evaluating the performance of different glaucoma classifiers. The performance of glaucoma classification amongst different glaucoma classifiers are integrated with imbalanced data strategies (e.g. SMOTE and class weight) is put in the comparison with our work, Table 2 tells the comparison result from different classifiers on ORIGA dataset. The Fig. 19 shows the corresponding ROC curves, along with AUC scores for glaucoma screening.

**Table 2:** Performance comparison of different classifiers on ORIGA dataset

Method	*Sensitivity*	*Specificity*	*ACC*	*AUC*
SVM	0.355	**0.972**	0.690	0.839
SVM + class weight	0.750	0.742	0.707	0.839
SVM + SMOTE	0.770	0.742	0.756	0.843
RF	0.500	0.908	0.719	0.837
RF + SMOTE	0.843	0.733	0.787	0.864
GDBT	0.500	0.917	0.726	0.832
GDBT+SMOTE	**0.857**	0.756	**0.806**	**0.878**

From the Table 2, it is concluded that the GDBT-based glaucoma classifier has achieved the best performance on *Sensitivity, ACC as well as AUC* evaluation criteria. Additionally, SVM, RF or GDBT classifier is capable of obtaining very high Specificity; comparatively, poor *Sensitivity* harboring a lot of glaucoma samples are put under misclassification as normalized when we conduct direct training on the classifiers upon the raw distribution of the imbalanced dataset. That displays the importance of the imbalanced data strategy. Further, the comparison between different methods utilizing SMOTE approach to cope with the imbalanced data are demonstratin that GDBT+SMOTE can respectively, manage 8.7 and 5% enhancement on *Sensitivity* and *ACC* than SVM + SMOTE and 1.4 and 1.9% than RF + SMOTE. In addition, GDBT+SMOTE attains best *AUC* score, compared with other m. Last but not least, GDBT classifier with SMOTE method achieves the best performance; therefore, GDBT+SMOTE is chosen as our last mode for our glaucoma screening work.

To further verify the performance of our final model, we evaluated GDBT+SMOTE classifier based on DRISHTI-GS1 [24] dataset with prediction accuracy. The glaucoma classification performance can be seen Table 3 and the result shows that GDBT+SMOTE classifier can produce stable and high accuracy.

**Table 3:** Performance test of GDBT+SMOTE classifier on DRISHTI-GS1 dataset

Classes	Counts	Predictions	Accuracy
Glaucoma	70	57	0.8143 (Sensitivity)
Non-glaucoma	31	22	0.7097 (Specificity)
All patients	101	79	0.7822

### Clinical setting assessments

As performance on benchmark data is no equality to those in the mobile App environment; therefore, we are joining hands with the Second Xiangya Hospital of Central South University for testing our Yanbao App. There are altogether 274 patients along with 648 fundus images were the targets which were achieved from the hospital’s eye clinics, our App prediction was made for the comparison with the results drawn by ophthalmologists. This research supports that our concluded diagnosis was established on the standard imaging as well as clinical parameters. The Table 4 illustrates the involved patients who are with or without glaucoma. In this research, the collection on what the ophthalmologist offered, including patient’s information such as gender, age, visiting date, and the diagnosis conclusions were conducted to analyze the eye illness with the patients’ contents. Other information associated with patients’ personal privacy was put under omission in the research. According to Table 4, it can be concluded that amidst the clinical backdrop, the no-glaucoma patient number is a lot larger than glaucoma patients. Most of the patients got over one retinal fundus images during the visit time. Taking into consideration our statistical results, 46 is the average age of all of the patients; the youngest one is merely 4 years old, and 90 is the oldest patient. Of all the glaucoma patients, the youngest one is 14 and 77 for the oldest one. Additionally, all the collected clinical data covers 7 years, lasting from October 14, 2010 to March 4, 2017. As for the 274 patients, the males are a little higher than women. The collected clinical cases above guarantee the sincerity and objectiveness featured by our performance test.

**Table 4:** Characteristics of the involved patients collected from hospital

Patient Property	Statistical results
Number of all patients	274
Number of glaucoma patients	104
Number of non-glaucoma patients	170
Number of all retinal fundus images	648
Average Age (range)	46 (4–90)
Data time range	2010.10.14–2017.3.4
Sex: Male: Female	155: 119
Eye: Left: Right	229: 309

**Fig. 20:**
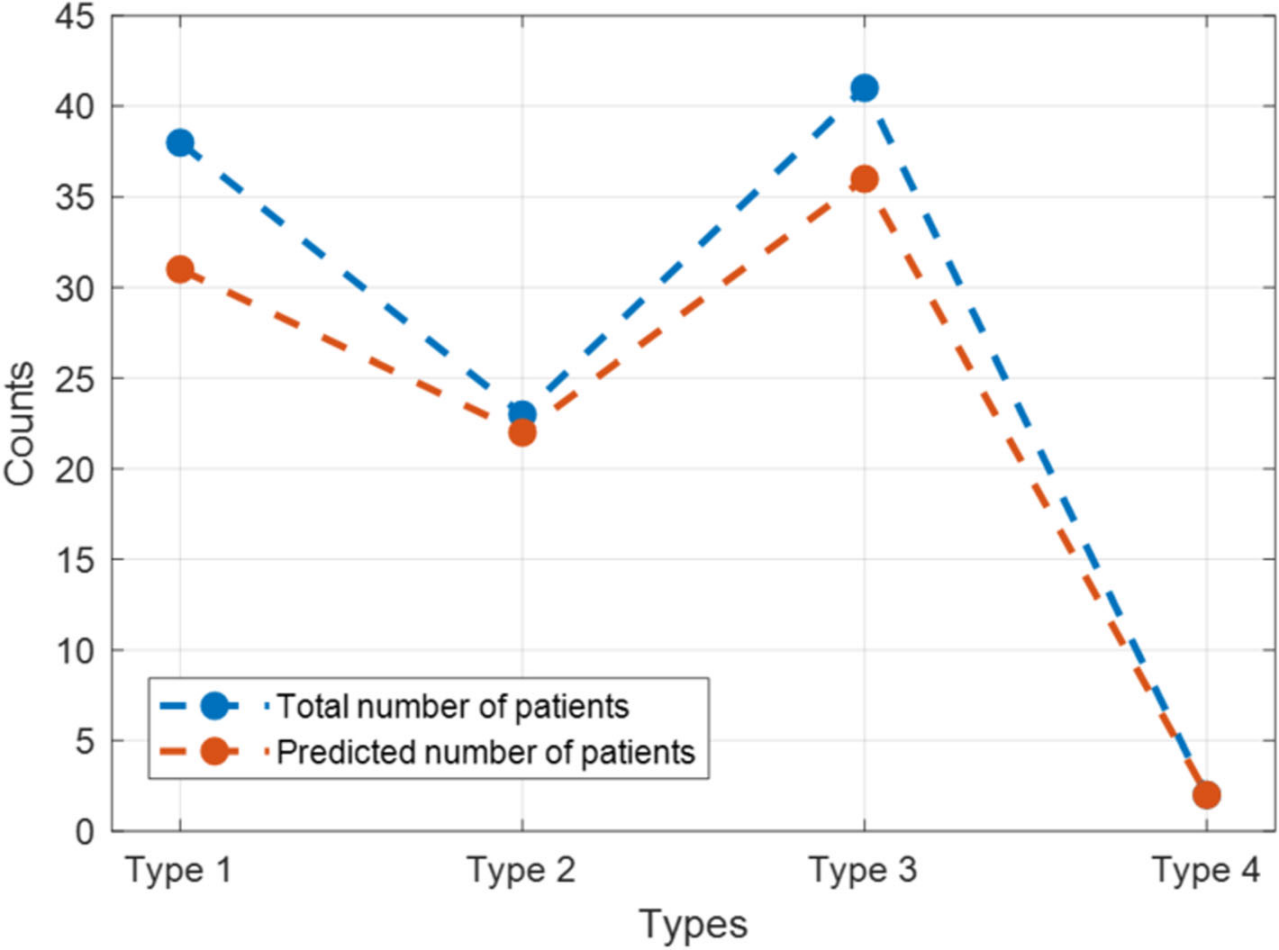
The performance of software predictions on different glaucoma types. Type 1: Primary angle-closure glaucoma. Type 2: Primary open-angle glaucoma. Type 3: Secondary glaucoma. Type 4: Congenital glaucoma

Following the further data cleaning, we the glaucoma disease is divided into four tiers: Primary angle-closure glaucoma and Primary open-angle glaucoma, Secondary glaucoma as well as Congenital glaucoma. Attention should be paid to different subclasses in secondary glaucoma from our collected data, like Hemorrhagic glaucoma and Pigmented glaucoma. To gain a convenient discussion, all the subclasses are deemed as secondary glaucoma in this research. Figure 20 demonstrates the comparison results from our developed App and ophthalmologist’s diagnosis on the four glaucoma main types.

Explicitly, for each of glaucoma type, our App is able to detect most of the cases by showing a comparatively high accuracy. Table 5 offers the consistent glaucoma detection accuracy. The Table helps to reach the deduction that our Yanbao App takes on similar performance on not only glaucoma but also non-glaucoma cases. Even the test cases number of glaucoma test is lot smaller value than non-glaucoma cases, the Secondary glaucoma harbors many subdivisions like Hemorrhagic glaucoma, Pigmented glaucoma and Malignant glaucoma. Experiment results demonstrate our App can conduct detection on most of the subclasses, which do not include Malignant glaucoma as the CDR-related and the ISNT-related characteristics takes on no evidence for Malignant glaucoma. Furthermore, also discovered that patients having failed in the test underwent glaucoma surgery or other eye illnesses (e.g. cataracts, and others), which might enhance the detection challenge and meanwhile introducing some baffling characteristics. Nonetheless, the big majority of glaucoma cases can achieve successful detection by our App. That means that the App merely assists doctors with glaucoma screening, but also benefit the users with assessing their own eyes condition.

**Table 5:** Prediction accuracy for the clinical test data

Classes	Counts	Predictions	Accuracy
Glaucoma	243	191	0.7860 (Sensitivity)
Non-glaucoma	405	310	0.7654 (Specificity)
All patients	648	501	0.7731

### Assessment of Yanbao app performance

For the measurement of the performance of our Yanbao App, three aspects are taken into consideration: (a) performance index testing, and (b) user experience assessment.

#### Test performance index

iOS smartphone iPhone 6 s Plus is used as the testing device to test the facility. The rear camera with 12 megapixels, is designed for fundus images, which can suit the diagnosis demands, in the combination with the relevant direct ophthalmoscope.

##### CPU and memory

**Fig. 21:**
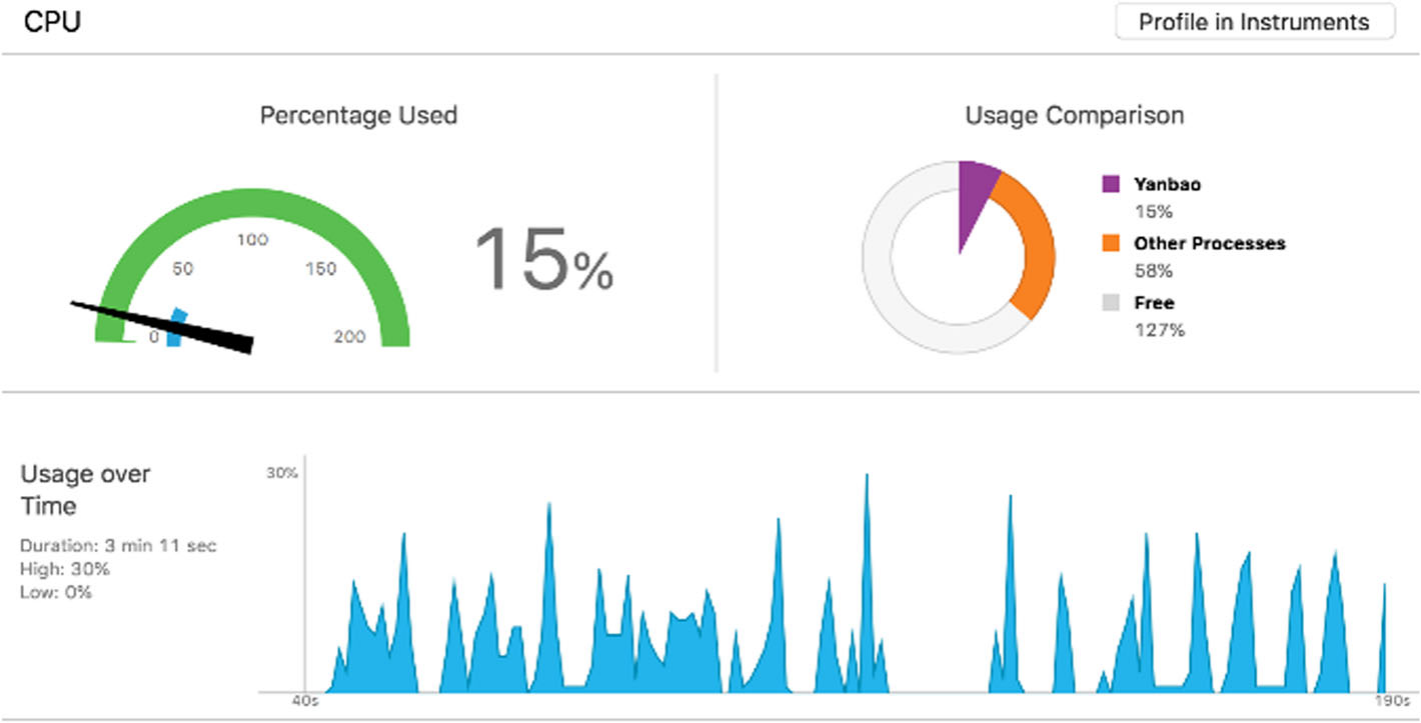
The usage of CPU on iPhone 6 s Plus while running our App

**Fig. 22:**
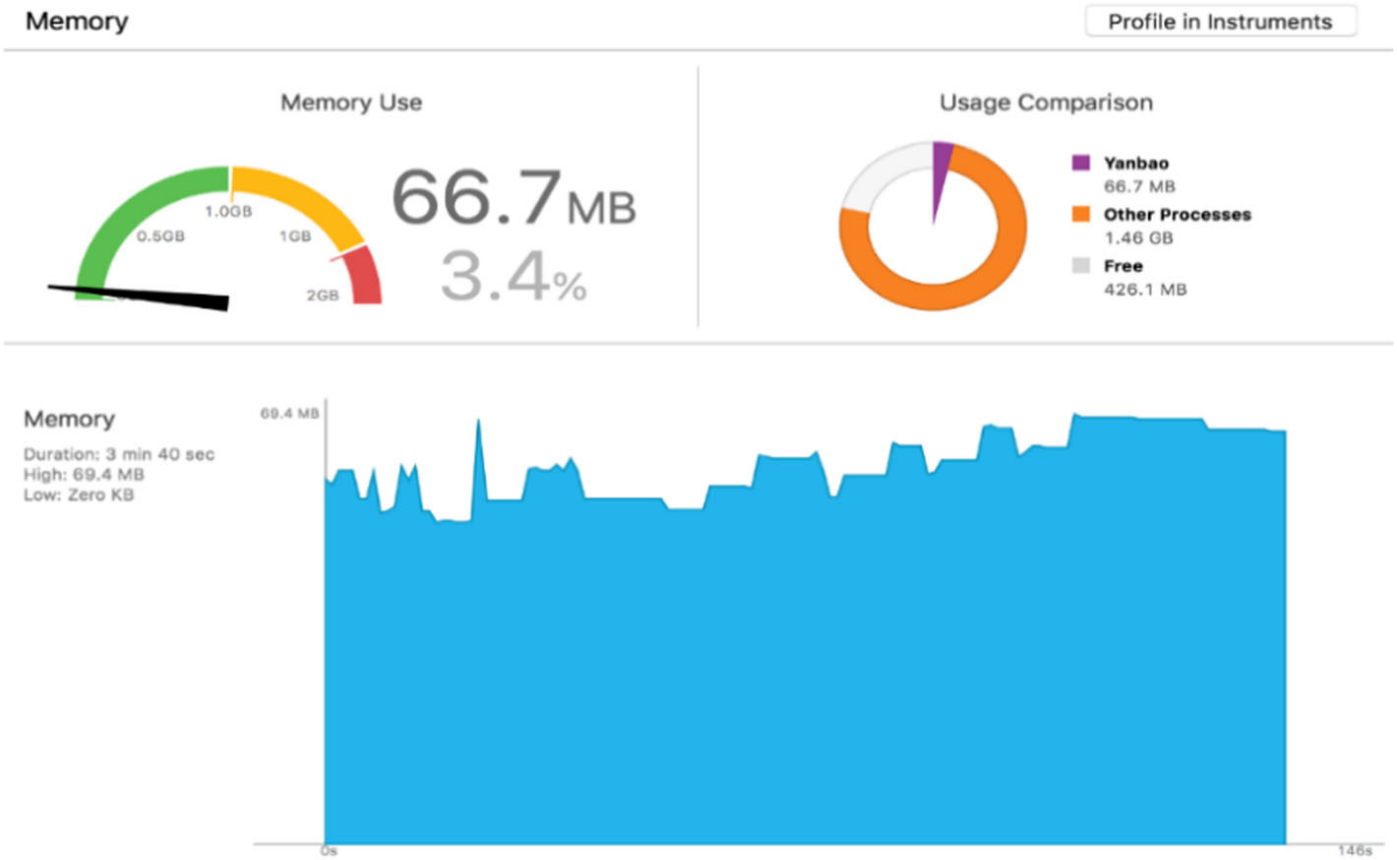
The usage of Memory on iPhone 6 s Plus while running our App

For obtaining accurate experimental results of operational characteristics, we have kept on uploading in-phone storage of multiple fundus images in several minutes, while checking the feedback. Figure 21 and Fig. 22 display the CPU and Memory usage on iPhone 6 s Plus in the meanwhile of the App operation.

As the figures demonstrates, of all the successive operations, CPU occupation amounts to 30%; which can merely last a short span and meanwhile exhibiting a large volume of fluctuations. Next, mainly as a result of multiple images with a coverage in our application and in feedback results as well. Memory consumption, which costs a peak value of 69.4 M, is featured by a comparatively small fluctuation. Additionally, GUI elements in iOS can be loaded with more memory storage. Except for overall smartphone operation, the memory consumption, which is at the level of less than 4%, will not affect the phone performance as a result of occupying a great deal of CPU as well as memory resources.

##### Response time

The APP response time have been tested, ranging from uploading file to demonstrating results, including the following:

- Shifting personal information as well as fundus images to JSON files.
- Sending out POST request and JSON files to cloud server.
- Receiving the testing results signaled from server.
- Processing the server feedback results and displaying on the screen.

**Fig. 23:**
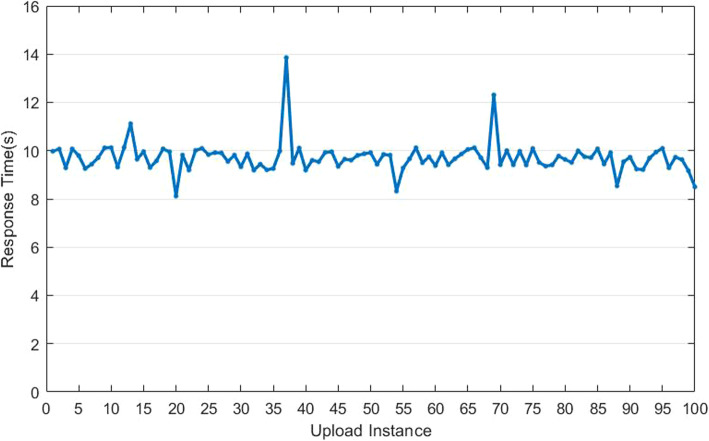
The response time of our App

To effectively test the response time, we did experiments in100 groups, chose fundus images of different specifications which need algorithm requirements. Figure 23 displaces the experimental results from 30 groups out of the experiments.

According to the results in Fig. 23, it is seen that 9.18 s is the average response time from 100 group experiments, with 0.82 s as the standard deviation. The reason for this long time is the limited computing resources of the cloud server as well as and the processing time of fundus images. In addition, as a result of some different delays depending on the network transition status, generally speaking, this app system harbors the operation with stability, reliability, feasibility and smoothness.

#### User experience evaluation

**Fig. 24:**
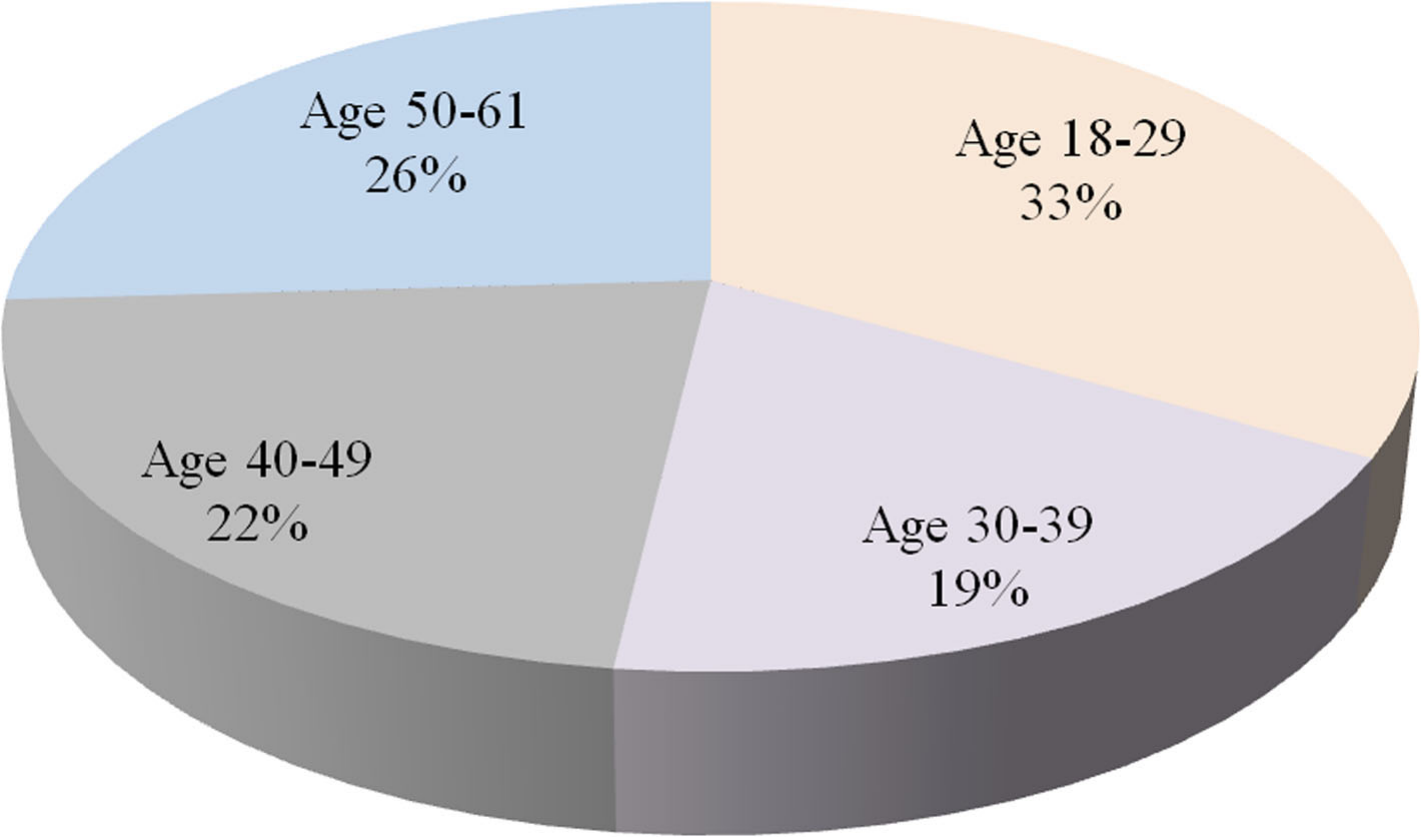
The age distribution of participants

To have an effective access to the user experience in the duration of applying Yanbao App, a range of usability tests and user research have been conducted in virtue of questionnaires. In detail, 27 people were invited here to join in the survey. The participators, whose average age was 31.6 (18–61), had no APP knowledge of the related information. The total survey group is made up of 16 males (59.3%), 11 females (40.7%) and 3 people (11.1%) who were working or studying in the area of the computer science. The age distribution of these participants can be seen in Fig. 24.

In our test, the basic ophthalmic knowledge was introduced to each of the participant, because the participants, who understand no fundus images, cannot operate the App. Besides, we informed that some fundus images have been stored in the phone. Next, a brief introduction was made to the participants on this application as well as its auxiliary diagnostic function. Attention should be paid that the users are not informed of the detailed ways of viewing the diagnostic information in this period. Besides, experimental results also prove our conclusions. All the participants can finish kinds of functional testing assignments independently. After the test, participants have to have the application-based questionnaire filled in and it is also a System Usability Scale (SUS) [24]. Previously, SUS used to be verified for its effectiveness of conduct App user test. Nowadays, SUS is widely applied around in systematic evaluation areas. SUS is made up of 10 items, and its rating result is classified as 1–5 levels. (1 refers to “strongly disagree”, 5 refers to “strongly agree”).

The SUS questionnaire questions are as follows.

1. In my opinion, I will employ this system more often.
2. I thought the system is of unnecessary complex.
3. So far as I know, it is an easy system to employ.
4. In my opinion, I am in need of a technician who is able to use the system to support me.
5. In my opinion, this system is featured by kinds of well-integrated functions.
6. My opinion is this system is equipped with too many inconstant aspects.
7. As far as I am concerned, I can imagine that most people would learn to use this system very quickly.
8. I stick to it that the system still includes some clumsy parts. .
9. I am convinced that I can use this system confidently.
10. I had to equip myself with a lot of knowledge previous to my getting on well with the system.

**Fig. 25:**
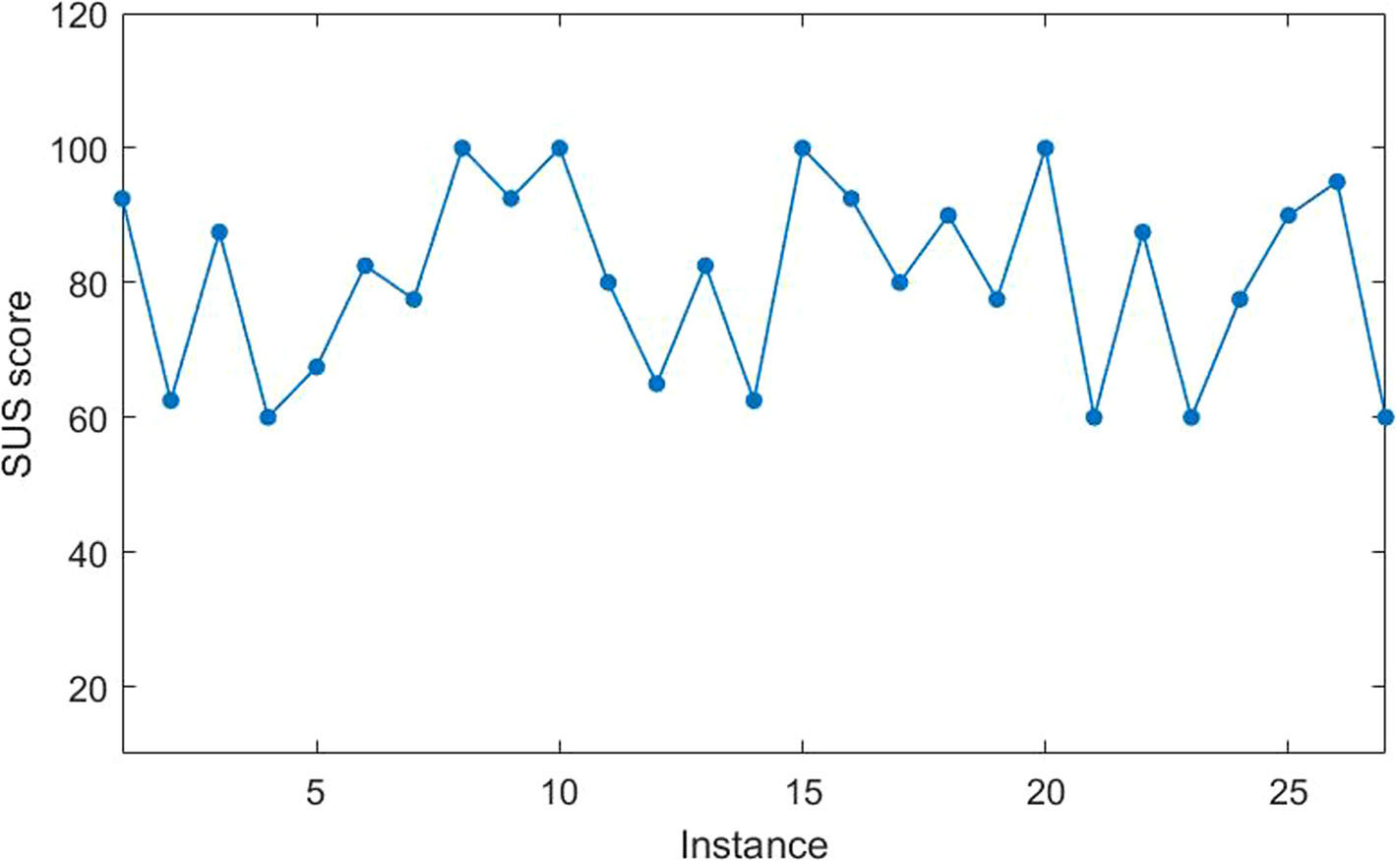
The usability scores of all the participants

In terms of the optionally direct items, the score marks that the scale position of minus 1. Next, as for the inverted items, the score is 5 minus scale position. At last, add all the items’ scores, and then multiply those by 2.5, the usability score will be achieved. As the basic score of each questionnaire is within the range of 0 ~ 40, the assessment score will be in the range of 0 to 100 after multiplying by 2.5. The whole staff of the participants has achieved success in finishing all the test tasks on the prerequisite of merely being informed of application usage plots and functions. Figure 25 signals more detailed aspects of each participant’s scores.

According to the Figure, the average score provided by the whole staff participants is 80.8, reflecting the high applicability of the App. Besides, the results also demonstrate that user interface’s design is rational for the users to comprehend its operation logic.

## Discussion

As of clinical setting test for Yanbao App, 274 patients were identified with 648 fundus images oriented with glaucoma classification evaluation. Diagnosis conclusion concluded by Ophthalmologists is considered to be a ground truth to assess the preciseness of our glaucoma screening App. In our experiment, 191 patients out of the 243 glaucoma patients were put under the screening at the accuracy rate of 0.7860 (Sensitivity). It is predicted that 310 out of 405 non-glaucoma patients were correct. The accuracy ratio was 0.7654 (specificity), which is lower than the Sensitivity in a small scale. Additionally, a person is able to see that 0.7731 is the total accuracy ration on the fundus images which were collected based on real clinical setting, nearly to to the test performance shown in on public dataset — ORIGA and DRISHTI-GS1. Thereby, it can be concluded that the proposed algorithm is characterized by strong robustness. It is suggested that Yanbao App should be employed as a strong tool to assist glaucoma screening.

Thanks to the mobile technology, the glaucoma diagnosis suggestions and evidences can be provided to users and doctors conveniently. The same is to other diseases. Nowadays, due to the increasing medical cost and global population aging, all the countries need better and more universal medical service, the medical area cries for transformation. We can clearly see that from patients to doctors, they pay more and more attention to the mobile technology and want to apply the technology to the treatment in order to cope with increasing challenges in this area. Besides, the transformation can also change of the human behavior mode and let more patient get prompt treatment at any time and any place. Thus, it can not only improve the treatment effect, but also reduce costs. In the transformation, smart mobile technology will play the important role. The universality of smart mobile technology and the ability of changing the human behavior caused by smart phone greatly drive the development of mobile medical treatment.

Mobile medical treatment involves various kinds of ways, which mainly include: passing on information to consumers to make healthier choice, and offering the patient information and initial diagnosis to doctors for reference to achieve the personalized medical treatment and improve the treatment effect. The factors of achieving the mobile medical treatment vision mainly have the following aspects: the first is the demand of mobile service. The maximum impetus of promoting the medical industry mobility is the basic demand from consumers and doctors. There have been wide demands on mobile service in many areas, like medical care. The second is the urgency of improving the medical system. The chronic disease and the relevant unhealthy lifestyle make the medical expense in developed countries in an unsustainable development state. While in developing countries, a large number of people can’t obtain medical service in time or suffer from diseases or death. Thus, the mobile solution is a must for them. The third is the successful experiments of mobile medical solution. Many experiments produce more and more evidence to support further application of mobile treatment. Just as the mobile solution of glaucoma diagnosis discussed in this paper, it can relieve the uneven distribution of medical resources effectively, alleviate the doctor’s work load, and reduce the rate of misdiagnosis at the same time. The same is to other mobile application in medical fields. These mobile applications all play the critical role for improving the human’s health conditions.

## Conclusions

The viability of introducing mobile technology in diagnosing diseases has been proved to be effective and efficient. Thus, an updated mobile App with the name Yanbao App is developed to allow users to conduct automatic real-time diagnosis glaucoma. It is practically applicable to lessen glaucoma patients’ burden by allowing them to be given good quality screening whenever and wherever. Thanks to the mobile technology, Yanbao App has started a new path to enhance the technicians’ working effectiveness and efficiency help strike medical resources balance so as to build excellent tiered medical services. Therefore, mobile technology or e-Health is likely to predict the blueprint of healthcare future.

## Data Availability

A description of the fundus image dataset and features included are provided in the manuscript. The ORIGA dataset and the DRISHTI-GS1 dataset are available from the first author upon reasonable requests. The real clinical dataset requires the approval of the hospital which these fundus images belongs to, thus the dataset cannot be made publicly available.

## Abbreviations

vertical CDR: Optic cup-to-disc ratio in vertical direction
area I disc ratio: Area ratio of I region to optic disc
thickness I: Thickness of I region
area I S ratio: Area ratio of I region to S region
thickness S N ratio: Thickness ratio of S region to N region
cup area: Optic cup area
area S disc ratio: Area ratio of S region to optic disc
area T disc ratio: Area ratio of T region to optic disc
